# Light-steerable locomotion using zero-elastic-energy modes

**DOI:** 10.1038/s41563-024-02026-4

**Published:** 2024-10-04

**Authors:** Zixuan Deng, Kai Li, Arri Priimagi, Hao Zeng

**Affiliations:** 1 Faculty of Engineering and Natural Sciences, Tampere University, Tampere, Finland; 2 Department of Civil Engineering, Anhui Jianzhu University, Hefei, China

**Keywords:** Polymers, Fluid dynamics

## Abstract

Driving synthetic materials out of equilibrium via dissipative mechanisms paves the way towards autonomous, self-sustained robotic motions. However, obtaining agile movement in diverse environments with dynamic steerability remains a challenge. Here we report a light-fuelled soft liquid crystal elastomer torus with self-sustained out-of-equilibrium movement. Under constant light excitation, the torus undergoes spontaneous rotation arising from the formation of zero-elastic-energy modes. By exploiting dynamic friction or drag, the zero-elastic-energy-mode-based locomotion direction can be optically controlled in various dry and fluid environments. We demonstrate the ability of the liquid crystal elastomer torus to laterally and vertically swim in the Stokes regime. The torus navigation can be extended to three-dimensional space with full steerability of the swimming direction. These results demonstrate the possibilities enabled by prestrained topological structures towards robotic functions of out-of-equilibrium soft matter.

## Abstract (web summary)

Zero-elastic-energy modes drive the self-sustained locomotion of a liquid crystal elastomer torus across various environments.

## Main

Biology serves as a profound source of inspiration for the development of synthetic materials that emulate the dynamic and adaptive behaviour of living organisms^1–3^. Insights drawn from the intricate mechanisms that sustain the functions of biological systems make it clear that the key characteristic for equipping inanimate materials with life-like properties is to drive them out of equilibrium^4–6^. This process paves the way for smart materials with autonomous, adaptive and interactive characteristics^7–9^. The out-of-equilibrium principle is particularly prominent in recent developments in soft actuators and soft robotics, in which self-regulating, feedback-driven interplay between structural deformation and energy dissipation may result in self-sustained, periodic motions under constant energy feed^10,11^. The energy input can be, for example, light^12,13^, heat^14^ or chemical reactions^15,16^, facilitating out-of-equilibrium functions such as locomotion^17–21^, object translocation^22^, homeostasis^23^ or signal transduction^24,25^.

Among the mechanisms used to drive soft materials into out-of-equilibrium states, zero-elastic-energy modes (ZEEMs) have emerged as particularly intriguing exemplars^26^. ZEEMs stem from the concept of zero-wavenumber, zero-frequency ‘hydrodynamic’ states in condensed matter in which continuous symmetry breaking emerges with no energy cost^27,28^. These modes manifest themselves in diverse scenarios, for example, quantum mechanics^29^, DNA topology^30^ and knots^31^. In the context of materials science, ZEEMs refer to mechanically frustrated systems with built-in mechanical strains, which can perform continuous motions under continuous external stimulation. Self-sustained, ZEEM-based motions have been demonstrated, for example, in nylon, polydimethylsiloxane (PDMS), hydrogel, fibreboids and liquid crystal elastomers (LCEs)^26,31–33^, offering a great platform for miniaturized, autonomous soft robotics^31,34,35^. A formidable challenge in ZEEMs, and in active soft matter in general, arises from the fact that motion parameters such as velocity and movement direction are poorly controllable when the system is driven far from equilibrium. Unidirectional self-sustained locomotion requires encoding asymmetry in either the geometry or deformability of the robotic construct, features that are difficult to adjust after fabrication^17,34^. However, dynamic steerability of self-sustained locomotion is vital for advanced soft-robotic functionalities such as active adaptation and autonomous interaction with changing surroundings^36^, and for the possibility of achieving autonomous swimming in the Stokes regime^37,38^.

Here we report an LCE torus that undergoes self-sustained rotation under constant exposure to light or heat. Exploiting dynamic friction or drag forces makes it possible to obtain light-steerable locomotion in various realms, including terrestrial, confined (on a thread or in a glass conduit) and fluidic environments. Echoing the vision raised by Purcell in his seminal lecture in 1976^37^, the toroidal structure exhibits light-steerable swimming in the Stokes regime, with Reynolds numbers (Re) of ∼0.0001.

## ZEEM system design

**Fig. 1:**
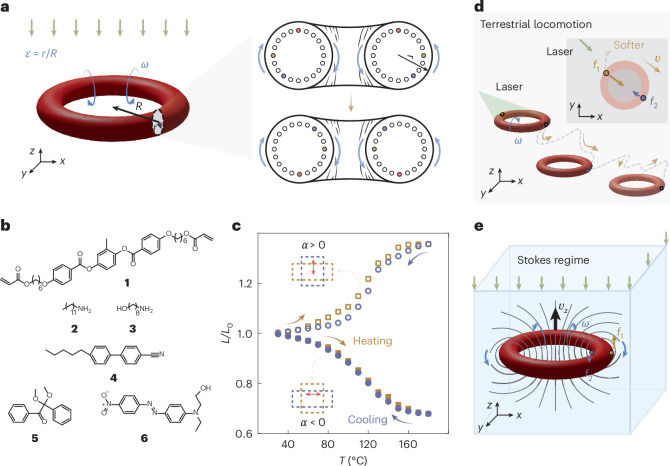
System concept of a light-steerable ZEEM torus. **a**, Left: schematics of the torus with radius *R*, made of a fibre with radius *r*. If *α* < 0, the torus inverts with an angular frequency *ω* under constant optical stimulation. The slenderness *ε* denotes the ratio between the fibre radius and the torus radius during excitation, *r*/*R*. Right: schematics of the cross-section of the torus during inversion. The upper and lower cartoons represent two instances (*t*_*n*_ and ${t}_{n+\frac{\uppi }{2}}$) during the inversion, with the marked coloured dots indicating unidirectional motion. **b**, Compounds used for the fabrication of light-responsive LCE fibres: **1**, LC crosslinker (RM82); **2** and **3**, amine chain extenders; **4**, non-polymerizable mesogen (5CB); **5**, photoinitiator; **6**, photothermal absorber (DR1). **c**, Heat-induced deformation during one heating–cooling cycle for LCE fibres with *α* < 0 (solid data points) and *α* > 0 (hollow data points). Insets: the corresponding dimensional change. The purple dashed box represents the pristine dimension and the orange dashed box indicates the deformed state. The double-headed arrow indicates the molecular alignment director. *L*_0_, original length of the sample; *L*, the measured length upon heating. **d**, Schematics of a torus with (*α* < 0) locomoting on a solid surface under oblique illumination. Inset: top view of the photothermally induced material softening at the contact interface, leading to distinct friction forces *f*_1_ and *f*_2_ with magnitude and direction represented by the arrows. **e**, Schematics of a light-responsive torus (*α* > 0) in the Stokes regime. Two distinct friction drags *f*_1_ and *f*_2_ are acting on the outer and inner sides of the active torus, respectively, with magnitude and direction represented by the scale and direction of the arrows. The grey lines represent the flow field around the rotating torus, inducing translational upwards motion with velocity *υ*_*z*_ towards the light field.

The ZEEM torus, initially demonstrated by Baumann et al., represents a mechanical structure in which the potential energy associated with rotational deformation is minimized or reduced to zero^26^. The torus can be driven out of equilibrium by a thermal stimulus, which continuously disrupts the spatiotemporal symmetry of the structure, leading to eversion or inversion. Figure 1a illustrates this motion and the resultant ZEEM inversion under uniform light illumination (photothermal heating). In response to a constant heat gradient induced by a hot plate or a light beam, the ZEEM torus becomes active and exhibits spontaneous inversion or eversion (Supplementary Fig. 1). Such autorotation is driven by the stimuli-induced deformability of the material, with the direction of rotation (inversion or eversion) dictated by the thermal expansion coefficient (*α*) of the material.

To fabricate the ZEEM toroidal structures, we use (photo)thermally responsive LCE fibres^39^. These fibres were prepared by injecting a monomer mixture (Fig. 1b) into 8-mm-diameter elastic silicone tubes. The mixture was oligomerized via an aza-Michael addition reaction between the acrylate groups of **1** and the primary amines **2** and **3**, using a slight excess of acrylates over amines (1.1:1 molar ratio)^40^. To reduce the viscosity of the polymerizable precursor, 50 wt% of non-polymerizable liquid crystal (LC) **4** was added to the mixture. The photopolymerization was conducted under mechanical strain to ensure monodomain alignment, as detailed in Methods and in Supplementary Fig. 2. The LCE fibres were placed on a hot plate for post-curing and to evaporate **4** before further use. The influence of the removal of **4** on thermal deformability is negligible, as shown in Supplementary Fig. 3.

Tube-based polymerization offers several advantages in controlling LC alignment prior to polymerization, and hence the thermal expansion coefficient of the resultant LCE. Unidirectional stretching induces alignment along the long axis of the fibre, resulting in a thermally driven, reversible contraction of ∼35% (Fig. 1c, *α* < 0). Conversely, when the tube is twisted 3 turns per cm during polymerization, LC alignment perpendicular to the long axis of the fibre is obtained, resulting in a reversible ∼35% expansion upon heating (Fig. 1c, *α* > 0). We ascribe this property to the synergy between the homeotropic anchoring of LCs onto the inner surface of the tube and the external twisting force. Hence, depending on polymerization conditions, the same material can yield either inverting or everting rotational motion. Further details on the influence of twisting on deformability, polarized optical microscopy images, heat-induced deformability and mechanical properties are given in Supplementary Figs. 4–7. To render the LCE fibres light sensitive, Disperse Red 1 (DR1), a light-absorbing molecule commonly used when devising photothermally actuated LCEs^22^, is incorporated by immersing an LCE fibre in DR1-containing isopropanol solution and letting DR1 diffuse into the fibre. The resultant red fibres display similar thermally driven deformability due to the photothermal effect, thereby ensuring sufficient work capacity (∼5 J g^−1^) for repeated deformations. Further details on the light-intensity-dependent strain, work capacity and reversibility of photoactuation under different loads are given in Supplementary Figs. 8 and 9. Eventually, the LCE fibres are looped by gluing together their two ends, yielding a light/heat-active ZEEM torus with rotation direction determined by the sign of *α*.

We utilize ZEEM autorotation for both self-sustained terrestrial motions and swimming. Figure 1d illustrates the principle of terrestrial locomotion, driven by friction asymmetry between the two sides of the active torus under oblique illumination. Figure 1e illustrates the hypothesis, initially proposed by Purcell^37^, of a self-rotating toroidal structure swimming at the low-Re limit. As we will show, these designs can be attained by using a ZEEM torus, the steerability of which is governed by incident light and the sign of *α*.

## Mechanistic study of ZEEMs in active toroidal structures

**Fig. 2:**
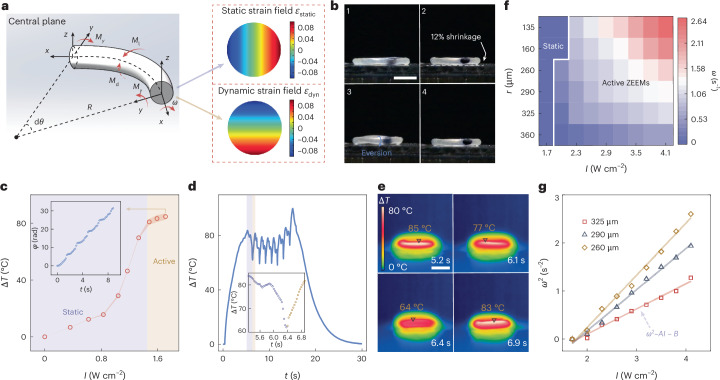
Mechanism of ZEEM formation in an active torus. **a**, Schematics of the driving torque and strain fields in the active torus. The strain field calculation is based on equations (2) and (8) in Supplementary Method 1.1.1. Dyn, dynamic. **b**, Photographs of an active torus rotating on a hot plate (120 °C). Scale bar, 2 mm. **c**, Photothermally induced temperature difference (Δ*T*, compared to room temperature, 23 °C) as a function of light intensity. Illumination direction is from the top down. Inset: cumulative rotational angle during several rotation cycles. **d**, Kinetics of Δ*T* over several inversion cycles under illumination. Inset: zoom-in to one cycle. **e**, Infrared images of an active torus under light irradiation at different stages within one inversion cycle. Irradiation conditions, 532 nm, 1.8 W cm^−2^. Scale bar, 2 mm. **f**, Colour map summarizing the activation threshold intensity and angular frequency of rotation (*ω*) of the ZEEMs for different fibre radii *r* on glycerol surface. **g**, *ω*^2^–*I* relation on theoretical fitting (solid line) of experimental results (symbols) for different fibre radii (260, 290, 325 μm). All demonstrated torus with negative thermal expansion coefficient. Each data point in **f** is presented as the mean value derived from three measured values. The same sample was measured repeatedly.

When an elastic fibre is closed to form a loop, topological prestrains naturally form, breaking the geometric symmetry along the cross-section of the torus (Fig. 2a, upper right). Once a heat gradient is generated (Fig. 2a, lower right), a torque *M*_d_ arises around the axis of the fibre due to the interplay between the mechanically built-in static strain field and the thermally driven dynamic strain field. When the power of the torque subdues the losses (*M*_d_ > *M*_l_), autorotation emerges (Fig. 2a, left). Further details on ZEEM mechanistics^26^, and calculation of the static and total elastic (static and dynamic) strain fields are given in Supplementary Method 1.1.1 and Supplementary Figs. 10 and 11. This behaviour can be confirmed by placing the torus on a hot plate (Fig. 2b and Supplementary Video 1). Further characterization of thermally driven eversion (*α* < 0) and inversion (*α* > 0) is given in Supplementary Figs. 12–14.

When illuminating the top surface of the LCE torus, the thermal gradient needed to activate the ZEEMs is provided by photothermal heating, which has a gradient direction opposite to that of direct heating from a hot plate. The maximum driving torque can be theoretically derived as ${M}_{\mathrm{d}}=\frac{\uppi E\alpha \beta \tau I\varepsilon {r}^{2}}{4}$, where *E* is Young’s modulus, *α* is the thermal expansion coefficient, *r* is the fibre radius, *ε* is the slenderness, *β* is a fitting parameter, *τ* is the characteristic time scale of thermal relaxation and *I* is the light intensity (see Supplementary Method 1.1.1 for derivation). For *α* < 0, a ZEEM becomes active once *I* reaches 1.5 W cm^−2^, corresponding to Δ*T* = 80 °C (Fig. 2c). The spontaneous rotation can be quantified by calculating the cumulative rotational angle, the nearly linear relationship of which indicates unidirectional rotation (Fig. 2c, inset). During the inversion, light-induced Δ*T* does not remain constant but fluctuates periodically in a sawtooth-like pattern with an approximate amplitude of 20 °C (Fig. 2d), as also shown by infrared imaging (Fig. 2e and Supplementary Video 2). Further details on temperature oscillation at different positions on the active torus are given in Supplementary Fig. 15. This echoes the mechanistic explanation of ZEEM formation: as *M*_d_ subdues the losses, the rotation causes the LCE surface originally exposed to light to rotate into shadow and cool down, while the cooler segment from the bottom rotates to the top and experiences photothermal heating. The continuous inversion ensures looped cycles, allowing the structure to self-sustain its rotational motion. Further details on the light-induced heating kinetics and temperature gradient profiles are given in Supplementary Figs. 16 and 17.

The light sensitivity of the active torus offers a convenient means to adjust its rotational behaviour because *ω* increases with *I* (Fig. 2f). When placed on a glycerol surface, the threshold to initiate inversion and to increase *ω* can be tuned by controlling the thickness of the LCE fibre: thinner samples tend to have a higher rotation threshold intensity, but once initiated, they rotate faster than thicker LCEs (Fig. 2f and Supplementary Video 3). Further details on the effect of slenderness, fibre radius and illumination angle on *ω* are given in Supplementary Figs. 18 and 19. For theoretical prediction of the intensity threshold and rotation speed, see equations (16) and (17) in Supplementary Method 1.1.2. Figure 2g shows the consistency between the recorded *ω* under different *I* (as given in Fig. 2f) and the theoretical prediction, *ω*^2^ = *AI* − *B*, where $A=\frac{{\uppi }{E|}\alpha |\beta }{4{G}^{\prime\prime}\tau }$, $B=\frac{1}{{\tau }^{2}}$, *τ* is the characteristic time for the thermal relaxation and *G*″ is the loss modulus. The mechanical testing results in Supplementary Fig. 20 indicate that internal losses contributed to the primary resistance torque during steady rotation. Details of the derivation are given in Supplementary Note 1.1.2. The photothermally driven rotation exhibits remarkable stability on a glycerol surface. Further details on the cumulative rotational angle and angular frequencies are given in Supplementary Fig. 21. It is worth noting that the rotation direction can be reversed if incident light of opposite direction or an LCE torus with *α* > 0 is used (Supplementary Figs. 22 and 23).

Non-equilibrium toroidal structures with self-perception and self-adaptation functionalities driven by thermal gradients have been demonstrated previously^9,35,41^. We build on these studies by showing that the motion of a self-sustained torus, fuelled by sitting on top of a hot plate, can also respond to an additional light field from the environment and change its motion accordingly. Supplementary Fig. 24 shows that the self-sustained eversion of a torus on a hot plate can be stopped with an additional light beam, and its translational motion can be terminated or redirected. In the following, we will implement an optical beam to induce both non-equilibrium ZEEM motion and light steering of the locomotion direction in different environments.

## ZEEMs enable multirealm locomotion

The Young’s modulus of an LCE decreases upon heating^42^, leading to temperature-dependent changes in the friction coefficient *μ*_s_ on different surfaces (Supplementary Fig. 25). A 3-fold increase in *μ*_s_ was observed upon 1.3 W cm^−2^ illumination. Consequently, oblique illumination results in different light-induced softening and friction coefficient on different sides of the torus, which can be leveraged for light-steerable terrestrial locomotion. A comparison between stochastically distributed motion on a hot plate and two-dimensional-steered, directed motion produced by a light beam is shown in Supplementary Figs. 26 and 27.

**Fig. 3:**
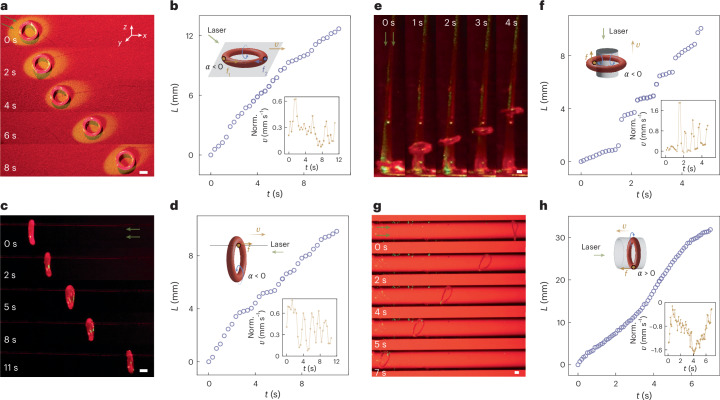
Multirealm locomotion. **a**–**d**, Snapshots of terrestrial movement of a light-sensitive torus on a dry surface (**a**) and on a thread (50 μm thick) (**c**), and the corresponding trajectories (**b**,**d**). Insets: corresponding force analysis and real-time normalized translational velocity. The magnitude and direction of the forces are represented by the scale and direction of the arrows. Normalized *υ* presents the real velocity in relation to the torus diameter. Irradiation conditions, 532 nm, 1.9 W cm^−2^. **e**–**h**, Snapshots of a light-sensitive torus moving in glycerol on a tapered pipette (**e**) and in a glass conduit (**g**), and the corresponding trajectories (**f**,**h**). Insets: analysis of corresponding force and real-time normalized translational velocity. Irradiation conditions, 532 nm, 7.3 W cm^−2^. Scale bars, 1 mm. Norm., normalized.

When the torus undergoes inversion under illumination (*α* < 0), both friction forces *f*_1_ and *f*_2_ act inwards, resulting in a force pointing towards the centre of the torus. Conversely, when the torus everts, the forces *f*_1_ and *f*_2_ acting on it point outwards, giving rise to a resultant force pointing outwards from the centre. Under oblique illumination with higher intensity on one side, the active torus rotation induces net movement away from the illumination direction if *α* < 0 (Fig. 3a and Supplementary Video 4) and towards the illumination direction if *α* > 0 (Supplementary Fig. 28). This is caused by distinct friction drags *f*_1_ and *f*_2_, acting on the torus: the side absorbing a higher photon flux becomes softer and experiences higher friction drag (*f*_1_) than the opposite side (see thermal imaging in Supplementary Fig. 29). The resultant asymmetric friction force gives rise to steady unidirectional motion (Fig. 3b), aligning with the direction of the higher friction drag (see force analysis in Fig. 3b, inset). The normalized translational velocity *υ* varies with *ε* and *r* (Supplementary Fig. 30). The terrestrial guided movement can also be conducted by hanging the torus on a thin thread and illuminating it horizontally, resulting in movement towards the excitation direction (Fig. 3c and Supplementary Video 5). In this case, the connection between the LCE torus and the thread is the only contact point providing static friction, which suffices for steady movement with no slipping (see force analysis in Fig. 3d, inset). Again, the movement speed depends on both *ε* and *r* (Supplementary Fig. 31). Examples of light-steered motion on other terrestrial surfaces, such as acrylic board, sandpaper, cloth, sand, and sloping and curved surfaces, are given in Supplementary Fig. 32.

In liquids with a high boiling point such as glycerol, the interfacial force plays a key role in governing the torus’s translocation. This behaviour is ascribed to the formation of bubbles due to photothermal heating; these bubbles readily accumulate at the interfaces, establishing irrevocable adhesion between the LCE and any surface encountered in the liquid medium^43^. Consequently, the LCE torus becomes bound to the solid surface, with the bubbles acting as spacers and as a capillary adhesive, thereby affecting the translocation behaviour of the torus. Figure 3e demonstrates the ability of a torus to climb a pipette wall by using spontaneous inversion (Supplementary Video 6). The corresponding trajectory exhibits a stepwise motion patten, indicating the influence of the encountered bubbles (Fig. 3f). The inner layer of the torus maintains close contact with the pipette, experiencing a friction drag acting towards the light field (see force analysis in Fig. 3f, inset). When submerged in non-polymerized PDMS, an intriguing feature emerges as initially proposed theoretically^44^. A rolling torus on a tapered pipette demonstrates the ability to rectify its translation direction, transitioning from rolling governed by friction forces to self-propulsion dominated by viscous drags as the aspect ratio between pipette radius and torus radius decreases. Further details of the locomotive modes upon changing pipette diameter are given in Supplementary Fig. 33. The locomotion can also be induced in more confined environments inside liquids, for example, in a 5 mm glass conduit. Here, an LCE torus with positive thermal coefficient (*α* > 0) is used, ensuring close contact with the inner surface of the conduit. The torus again moves towards the light source, with its outer ring interacting with the glass passage filled with viscous glycerol (Fig. 3g,h and Supplementary Video 7). As the ZEEM-enabled eversion occurs, a friction drag *f* is imposed on the outer layer, acting in the direction pointing towards the light source (see force analysis in Fig. 3h, inset). Further details and other examples on light-steered locomotion in glycerol are given in Supplementary Figs. 34–36.

## Light-steerable swimming in the Stokes regime

Although Purcell proposed his scallop theorem decades ago^37^, the formidable challenge of creating an out-of-equilibrium toroidal swimmer in the low-Re limit, for example, in the Stokes regime (Re ≪ 0.1), is yet to be resolved. Strategies for endowing swimmers with increased degrees of freedom sacrifice swimming performance because backflow occurs during the recovery stroke, mitigating the positive flow propelled by the power stroke^45,46^. Purcell envisaged an imaginary toroidal topology for enhanced swimming efficiency, which we demonstrate here with our ZEEM torus^37^.

**Fig. 4:**
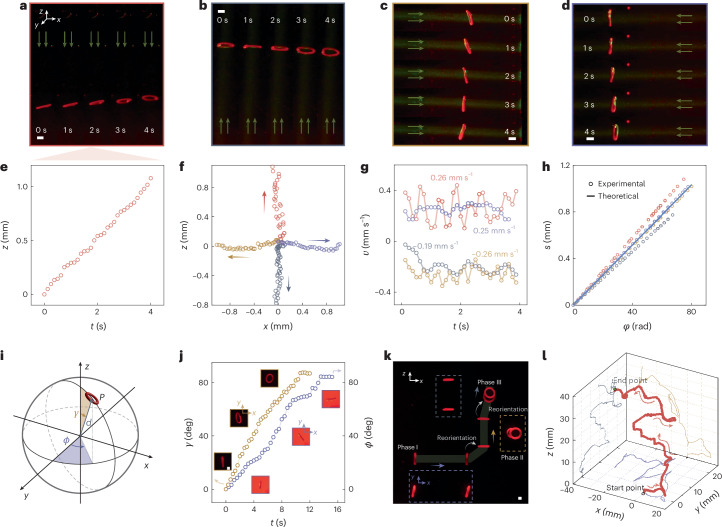
Light-steerable swimming in the Stokes regime. **a**–**d**, Snapshots of an active torus swimming in non-polymerized PDMS in upwards (**a**), downwards (**b**), leftwards (**c**) and rightwards (**d**) directions. **e**, Trajectory of the upwards movement. **f**, Trajectories for the four movement direction scenarios in the 4 s irradiation period. The coloured arrows indicate the movement direction, with relative swimming velocity represented by the scale of the arrows. **g**, Corresponding translational velocity in the *x*–*z* plane. The values given suggest the average velocity, while the sign represents the movement direction. **h**, Relation between absolute translational displacement *s* and rotational angle *φ* for the four movement directions, together with theoretical prediction (solid line). **i**, Orientation of a torus in the spherical coordinate system. *γ*, polar angle; *ϕ*, azimuthal angle; *d*, radial line. **j**, Temporal evolution of the two angular variables upon optical reorientation. Insets: photographs of the torus altering its facet at different stages. The images in solid orange boxes represent changes of angle *γ* observed in the *x*–*y* plane; the images in purple boxes represent changes of angle *ϕ* observed in the *x*–*y* plane. **k**, Sequential snapshots of an active torus translating in three dimensions, observed from the anterior view in the *x*–*z* plane. The enveloped images in the dashed boxes represent the dorsal view in the *x*–*y* plane. **l**, Trajectory of an active torus navigating through three-dimensional space (red curve), with projections onto the *x*–*y*, *x*–*z* and *y*–*z* planes. Irradiation conditions, 532 nm, 7.3 W cm^−2^. Scale bars, 1 mm.

To obtain an operating environment in the Stokes regime, an optically transparent, viscous PDMS (viscosity, 5.5 Pa s) elastomer base was used, resulting in a Re of ~0.0001. When the PDMS-immersed torus (*α* > 0) is illuminated, its movement trajectory orchestrates the direction of the imposed light field. Consequently, the ZEEM torus can undergo autonomous swimming to any direction, determined by the illumination direction (Fig. 4a–d and Supplementary Video 8). Taking upwards swimming as an example, the cumulative distance over a few rotation cycles collects linearly along the *z* axis, suggesting steady upward translocation (Fig. 4e). When the torus undergoes eversion (*α* > 0), two viscous drags *f*_1_ and *f*_2_ act on the surfaces in opposite directions. The force *f*_1_ on the outer surface points towards the light source, exhibiting larger value due to the bigger surface area, thereby causing the torus to propel itself towards the light source. In the Stokes regime, because the viscosity dominates the mobility, the active torus experiences distinct viscous drag forces on its outer and inner layers due to their different surface areas, which leads to a unidirectional locomotion dictated by the direction of the drag force imposed on the outer layer (see the force analysis in Fig. 1e). The reverse effect can be observed for an active torus with a negative thermal expansion coefficient (*α* < 0), which swims in the opposite direction compared to the movement directions depicted in Fig. 4 under identical illumination conditions (Supplementary Fig. 37).

Four different trajectories normalized to the *x*–*z* plane are shown in Fig. 4f and the corresponding swimming speeds are given in Fig. 4g. The leftwards and rightwards swimming speeds are practically identical, but upward swimming outperforms downward swimming. This can be attributed to heat convection parallel to the *z* axis, which assists the upwards motion while obstructing the downwards motion, but has no effect on the sideways motions. Further details on the swimming trajectories and velocities are given in Supplementary Figs. 38 and 39. Figure 4h shows the absolute translational displacement *s* in relation to the rotational angle *φ* in all directions. The revealed linear *s*–*φ* dependency is supported by the theoretical prediction $\frac{s}{\varphi }=\zeta \frac{r\varepsilon }{2}\left(\mathrm{ln}\frac{8}{\varepsilon }-\frac{1}{2}\right)=\mathrm{constant}$ (where *ζ* = 0.36 is the fluidic sliding coefficient) for Stokes regime swimmers (see Supplementary Method 1.2 for derivation details). Further details on the rotational angle *φ* in the four scenarios are given in Supplementary Fig. 40.

When exposed to an inhomogeneous light intensity pattern, our toroidal swimmer tends to adjust its excitation facet by inclining until it experiences uniform light intensity, with the side experiencing higher light intensity serving as the stator, and the opposing side revolving around it (Supplementary Video 9). We ascribe this behaviour to a photothermally induced viscosity change and the resultant uneven drag distribution around the torus. This phenomenon can be exploited to manipulate the swimming direction of the torus, as indicated by the polar angle *γ* and the azimuthal angle *ϕ* in the spherical coordinate system (Fig. 4i). The kinetics of changing the orientation angles is showcased in Fig. 4j, indicating an optically driven reorientation of about 7° s^−1^. Figure 4k shows the directional swimming combined with intermittent reorientation to the orthogonal directions. First, the torus travels along the *x* axis for 15 mm (phase I). Second, the torus is optically reoriented to travel along the *z* axis (phase II). Third, the direction is again changed to follow the *y*-axis direction (phase III). By controlling the position of the excitation beam, a three-dimensional swimming course can be achieved, as exemplified by the trajectory shown in Fig. 4l and Supplementary Video 10.

## Discussion

The continuously breaking spatiotemporal symmetry of the active toroidal structure enables it to attain net propulsion in the low-Re limit, which combined with limited liquid sliding can greatly enhance swimming efficiency because the rotation is unidirectional^38^. Unlike strategies employed in other artificial microswimmers, for instance, Purcell’s three-link swimmer^45,46^ or the ‘pushmepullyou’ swimmer^47^, the minimized potential energy change associated with rotational deformation in the ZEEM toroidal swimmer presents a particularly promising platform for enhanced swimming performance in the Stokes regime. Details of Re calculation and particle image velocimetry analysis are given in Supplementary Note 1.3 and Supplementary Fig. 41, respectively.

The elastomeric torus provides a generic approach to achieve ZEEMs, but the possibilities are not limited to toroidal geometry alone. Plentiful configurations can be conceived such as Möbius strips^48^, edge-crumpled sheets^49^ and supercoils with slithering behaviour^30^, among others. This generalized concept can also be applied to other responsive materials, such as hydrogels, shape memory materials and piezoelectric materials.

## Conclusions

We present a light-fuelled, millimetre-sized elastomeric torus that exhibits self-sustained rotational and translational motion under constant light illumination. The active torus, made of thermally responsive LCE fibre, undergoes spontaneous rotation under visible-light excitation, due to formation of global ZEEMs that drive the self-sustained motion. The rotation direction (eversion or inversion) is dictated by the thermal expansion coefficient of the LCE along the long axis of the fibre, which can be preset to be either positive or negative during the fabrication process, based on the programmability of LCE alignment. By exploiting the dynamic friction or drag forces, a myriad of locomotive behaviours in various realms are attained, including terrestrial, confined (on thread and in glass conduit) and fluid environments. Most importantly, untethered light-driven toroidal swimmers in the Stokes regime (Re ≈ 0.0001), as proposed by Purcell in 1976^37^, have been achieved. We observed a dynamic self-reversal of locomotion direction on a tapered pipette under low-Re conditions, as predicted by Kulić and co-workers^44^. By pushing the boundaries of life-like synthetic materials, this work paves the way for the design of non-equilibrium soft locomotors with light steerability and possibilities for controlled robotic swimming in the Stokes regime.

## Methods

### Materials in brief

1,4-Bis-[4-(6-acryloyloxyhexyloxy) benzoyloxy]-2-methylbenzene (99%, RM82) was obtained from SYNTHON Chemicals. 8-Amino-1-octanol, dodecylamine and 4-cyano-4′-*n*-pentylbiphenyl (5CB) were obtained from TCI. 2,2-Dimethoxy-2-phenylacetophenone and glycerol solution (86–89%) were obtained from Sigma-Aldrich. DR1 and PDMS elastomer base (Sylgard 184) were obtained from Merck. Silicone tubes were obtained from VWR. All chemicals were used as received.

### Sample fabrication

The LC mixture was prepared by mixing 0.22 mmol RM82, 0.1 mmol 8-amino-1-octanol, 0.1 mmol dodecylamine, 50 wt% 5CB and 2.5 wt% of 2,2-dimethoxy-2-phenylacetophenone at 80 °C. The mixture was injected into an elastic silicone tube at 80 °C and maintained for 10 min before cooling down to 45 °C (1 °C min^−1^). The sample was kept in the oven for 24 h at 45 °C to allow aza-Michael addition reaction for oligomerization. Then, mechanical forces (stretching or twisting) were applied on the sample before polymerization with ultraviolet light (365 nm, 180 mW cm^−2^, 30 min). After polymerization, the tube was removed in isopropanol, and the obtained LCE fibre was placed on a hot plate at 120 °C to complete the curing process and to evaporate 5CB before being transferred to a DR1/isopropanol solution for dyeing (see Supplementary Fig. 42 for absorption spectra). The LCE fibre was glued with superglue to form the ZEEM torus.

### Optical characterization

Absorption spectra were measured with a custom-modified ultraviolet–visible spectrophotometer (Cary 60 UV–vis, Agilent Technologies). Photographs and movies were captured with a Canon 5D Mark III camera with a 100 mm lens. Thermal images were recorded with an infrared camera (FLIR T420BX) with a close-up 2× lens. A continuous-wave laser (532 nm, 2 W, Roithner) was used for light excitation.

### Mechanical characterization

Dynamic mechanical analysis tests were performed in the temperature range from −18 °C to 110 °C using a Modular Compact Rheometer MCR-702e (Anton Paar) in tension mode. A fibre sample of diameter 600 μm was attached to the measuring clamps with a 12 mm free length. The tension experiment was run in stress-control mode at 1 Hz with 0.04 MPa oscillating amplitude and 0.044 MPa static stress. The temperature was increased at 2 °C min^−1^.

### Data analysis

The movement was recorded, and quantitative data were extracted from the movie by using video analysis software (Tracker). The fluid field evolution was analysed with particle image velocimetry software in Matlab^50^.

### Modelling

The calculation data were acquired based on the modelling and theoretical formulation, which are provided in Supplementary Methods. The geometrical and physical parameters are summarized in Supplementary Figs. 10 and 11. The assumptions are also discussed in Supplementary Methods. For details of the calculations, see Supplementary Methods.

## Online content

Any methods, additional references, Nature Portfolio reporting summaries, source data, extended data, supplementary information, acknowledgements, peer review information; details of author contributions and competing interests; and statements of data and code availability are available at 10.1038/s41563-024-02026-4.

## Supplementary information

Supplementary Information Supplementary methods for modelling, Figs. 1–42 and captions for Supplementary Videos 1–10.

Supplementary Video 1 Thermally driven everting ZEEM torus.

Supplementary Video 2 Infrared imaging of optically driven inverting ZEEM torus under top-down illumination.

Supplementary Video 3 Optically driven rotation of ZEEM torus on an air–glycerol interface.

Supplementary Video 4 Terrestrial movement of ZEEM torus walking on land.

Supplementary Video 5 Terrestrial movement of ZEEM torus crawling on a thread.

Supplementary Video 6 ZEEM torus climbing on a pipette in glycerol.

Supplementary Video 7 ZEEM torus moving in a glass conduit filled with glycerol.

Supplementary Video 8 Steerable untethered swimming of ZEEM torus in the Stokes regime.

Supplementary Video 9 Optical reorientation in the Stokes regime.

Supplementary Video 10 Untethered swimming through three-dimensional space in the Stokes regime.

## Data Availability

The raw data generated in this study have been deposited in Fairdata IDA online storage space at 10.23729/67dabfbb-80ac-498f-9583-0405b71ad169. Data are available from the corresponding author on request.
