# Supplementary material for: Light-steerable locomotion using zero-elastic-energy modes
Source: Nat Mater. 2024 Oct 4;23(12):1728–35. doi: 10.1038/s41563-024-02026-4 (PMC11599032; doi:10.1038/s41563-024-02026-4)
Supplement: Supplementary file 1 — Supplementary methods for modelling, Figs. 1–42 and captions for Supplementary Videos 1–10. [file 41563_2024_2026_MOESM1_ESM.pdf]

nature materials

Supplementary information https://doi.org/10.1038/s41563-024-02026-4

Light-steerable locomotion using zero-elastic-energy modes

In the format provided by the authors and unedited

Table of contents:

1. Supplementary Methods for modeling.

1.1 Mechanism of active torus on a flat surface.

1.2 Self-swimming of active torus in Stokes fluid.

1.3 Calculation of Reynolds number.

2. Supplementary Figures 1-42.

3. Captions for Supplementary Videos 1-10.

Supplementary Notes.

1. Supplementary Methods for modeling.

1.1 Mechanism of active torus on a flat surface.

1.1.1 Driving torque.

Here, we provide a brief overview of the theoretical framework, referencing the work in Ref. 1. The radius r of fiber is assumed to be much smaller than the curvature radius R of torus, and the deformation of torus is assumed to be small. The curvature radius of torus is R, and the radius of fiber is r. For steady rotation of torus, the elastic strain on the cross-section can be written as[1]

$$\varepsilon_e(\xi,\theta) = \varepsilon_{static}(\xi,\theta) + \bar{\varepsilon} + \varepsilon_T(\xi,\theta), \tag{1}$$

where, the static strain field due to the bending of the fiber is[1]

$$\varepsilon_{static}(\xi,\theta) = -\frac{r}{R}\sin\theta, \tag{2}$$

$\bar{\varepsilon}$ is related to axial compression/elongation, and the thermal strain due to uniform axial thermal expansion/shortening is assumed to be linearly related to the temperature field as

$$\varepsilon_T(\xi,\theta) = \alpha T(\xi,\theta), \tag{3}$$

where, α is the linear thermal expansion coefficient. For simplicity, the temperature field is assumed to be[1],

$$T(\xi,\theta) = T_0 + T_{cos}\frac{\xi}{r}\cos\theta + T_{sin}\frac{\xi}{r}\sin\theta. \tag{4}$$

In Eq. (4), the two main temperature modes T_{cos} and T_{sin} are considered, which can be determined by the following governing equations[1],

$$\dot{T}_{sin} = -\frac{T_{sin}}{\tau} - \omega T_{cos},\ \dot{T}_{cos} = p - \frac{T_{cos}}{\tau} + \omega T_{sin}, \tag{5}$$

where, τ is the characteristic time scale of the thermal relaxation, ω is the angular frequency, and the pumping rate p is assumed to be proportional to the light intensity I, i.e. $p = \beta I$, with β being

the fitting parameter. For steady rotation of the active torus, it can be obtained from Eq. (5) that

$$T_{cos} = \frac{\beta\tau I}{1+\tau^2\omega^2}, T_{sin} = -\frac{\beta\tau^2\omega I}{1+\tau^2\omega^2}. \quad (6)$$

Combining Eqs. (1)-(5) leads to

$$\varepsilon_e(\xi,\theta) = \varepsilon_{static}(\xi,\theta) + \varepsilon_{dyn}(\xi,\theta), \quad (7)$$

in which, the dynamic strain field is related to the angular frequency as

$$\varepsilon_{dyn}(\xi,\theta) = -\frac{\alpha\beta\tau I}{1+\tau^2\omega^2}\frac{\xi}{r}\cos\theta + \frac{\alpha\beta\tau^2\omega I}{1+\tau^2\omega^2}\frac{\xi}{r}\sin\theta. \quad (8)$$

It is noted that $\bar{\varepsilon} = \alpha T_0$ during deriving Eq. (7) when the axial deformation from the frictional force is ignored. From Eqs. (2) and (8), the static strain field and dynamic strain field can be plotted, as shown in Supplementary Figures 10 and 11.

For simplicity, it is also assumed that the material is linearly elastic. Then, the normal stress on a cross section of the torus is

$$\sigma(\xi,\theta) = E\varepsilon_e(\xi,\theta), \quad (9)$$

where, E is the Young's modulus. The bending torque about the x axis can be calculated as

$$M_x = \int_0^r \int_0^{2\pi} \sigma(\xi,\theta)\xi\cos\theta\,\xi d\xi d\theta. \quad (10)$$

Selecting an arbitrary segment from the torus as shown in Fig. 2(a), we can obtain the driving torque (normalized by the arc length) applied to the segment as[2],

$$M_d = \frac{M_x}{R}. \quad (11)$$

By combining Eqs. (7)-(11), the driving torque can be expressed as

$$M_d = \frac{\pi E\alpha\beta\tau}{4}\frac{I\varepsilon r^2}{1+\tau^2\omega^2}, \quad (12)$$

where, $\varepsilon = \frac{r}{R}$, denotes the slenderness of the torus. From Eq. (12), the driving torque attains the maximum when the torus stays static, which is

$$M_d^{max} = \frac{\pi E\alpha\beta\tau}{4} I\varepsilon r^2. \quad (13)$$

If the maximum driving torque is less than the resistance torque, the torus cannot rotate and remains stationary.

1.1.2 Angular frequency.

During the steady rotation, the resistance torque primarily comes from the internal losses of the torus, which is much bigger than the external resistance torque caused by the sliding resistance force of the supporting surface[1]. The dissipative torque from internal losses can be estimated to be proportional to the slenderness of the torus[1]. Then, for the torus rotating on a flat surface, the resistance torque per unit arc length of the torus can be given as[1]

$$M_l = G''\varepsilon r^2, \quad (14)$$

where, G'' is the loss modulus of torus.

When the maximum driving torque is greater than the resistance torque, the torus starts to rotate. The angular frequency of rotation is determined by the balance between the driving moment and the resistance moment, i.e. $M_l = M_d$. By inserting Eqs. (12) and (14), the equilibrium equation leads to

$$\omega = \frac{1}{\tau}\sqrt{CI - 1}, \quad (15)$$

where, $C = \frac{\pi E|\alpha|\beta\tau}{4G''}$. From Eq. (15), it can be seen that the angular frequency increases with the increases of light intensity, which is consistent with the physical intuition.

Eq. (15) can also be written as

$$\omega^2 = AI - B, \quad (16)$$

where, $A = \frac{\pi E|\alpha|\beta}{4G''\tau}, B = \frac{1}{\tau^2}$. From Eq. (16), the angular frequency may increase with the decrease of the fiber radius, considering that decreasing fiber radius leads to decreasing τ and increasing A.

By setting $\omega = 0$ in Eq. (16), we can obtain the critical light intensity for triggering the rotation,

$$I_{\text{crit}} = \frac{4G''}{\pi E|\alpha|\beta\tau}. \tag{17}$$

In Eq. (17), the critical light intensity decreases with increasing fiber radius, for τ increases with increasing fiber radius. The result is consistent with the experimental results in Fig. 2f.

1.2. Self-swimming of active torus in Stokes fluid.

As shown in Fig. 4, under the action of the light-driven torque M_d and external payload P (gravity for the free torus), the torus rotates and translates in fluids. The torus is also subject to the viscous force F_{fluid} and the torque M_{fluid} from the surrounding fluid. For steady swimming, the force F_{fluid}, external force P, the torque M_{fluid} , and the driving toque are balanced. Following the related work[3], the F_{fluid} and the toque M_{fluid} can be given, and the equilibrium equation of the swimming torus are derived as

$$P = 4\pi^2\mu\left(-\frac{2R}{ln\frac{8}{\varepsilon}+\frac{1}{2}}v + r^2\frac{ln\frac{8}{\varepsilon}-\frac{1}{2}}{ln\frac{8}{\varepsilon}+\frac{1}{2}}\omega\right), \tag{18}$$

$$M_d = 2\pi\mu r\left(-\frac{ln\frac{8}{\varepsilon}-\frac{1}{2}}{ln\frac{8}{\varepsilon}+\frac{1}{2}}v + 2R\omega\right), \tag{19}$$

where μ is the viscosity of the fluid.

From Eq. (18), the relation between the translation velocity and the rotation angular frequency can be derived as

$$v = \frac{\omega r\varepsilon}{2}\left(ln\frac{8}{\varepsilon} - \frac{1}{2}\right) - \frac{P\varepsilon}{8r\pi^2\mu}\left(ln\frac{8}{\varepsilon} + \frac{1}{2}\right). \tag{20}$$

For the case where P is negligible, Eq. (20) is degenerated to

$$v = \frac{r\varepsilon}{2}\left(ln\frac{8}{\varepsilon} - \frac{1}{2}\right)\omega. \tag{21}$$

Taking into account the sliding effect inside the liquid, the rotation angle φ is proportional to the translation displacement s, i.e.

$$\frac{s}{\varphi} = \zeta \frac{r\varepsilon}{2}\left(ln\frac{8}{\varepsilon} - \frac{1}{2}\right). \tag{22}$$

where ζ is the fluidic sliding coefficient. It is shown from Eq. (22) that the ratio of s to φ only depends on the geometric parameters of fiber radius and slenderness.

Additionally, combining Eqs. (12), (19) and (21) leads to

$$\mathrm{v} + \frac{4\tau^2}{\mathrm{r}^2\varepsilon^2(\ln\frac{8}{\varepsilon}-\frac{1}{2})^2}\mathrm{v}^3 = \frac{\mathrm{E}\alpha\beta\tau}{8\mu}\frac{\mathrm{I}\varepsilon\mathrm{r}}{\left(\frac{\ln\frac{8}{\varepsilon}-\frac{1}{2}}{\ln\frac{8}{\varepsilon}+\frac{1}{2}}+\frac{4}{\varepsilon^2\left(\ln\frac{8}{\varepsilon}-\frac{1}{2}\right)}\right)}. \tag{23}$$

By solving Eq. (23), the swimming velocity of the torus in the low Reynolds limit can be obtained. It can be seen from Eq. (23) that the swimming velocity increases with the increasing light intensity, slenderness or thickness of torus. These results are consistent with the experimental results in Supplementary Figure 39.

1.3 Calculation of Reynolds number

The Reynolds number (Re) is defined as the ratio of inertial forces to viscous forces, $Re = \frac{\rho D v}{\mu}$. In the PDMS base (at 25 °C, the dynamic viscosity $\mu = 5.5$ Pa s and the density $\rho = 1.1$ g cm^{-3}), for active torus with diameter $D = 2.2$ mm that swims with a velocity of 0.26 mm s^{-1}, $Re \approx 10^{-4}$, suggesting swimming in Stokes regime where viscous drag governs the motion of the torus.

2. Supplementary Figures.

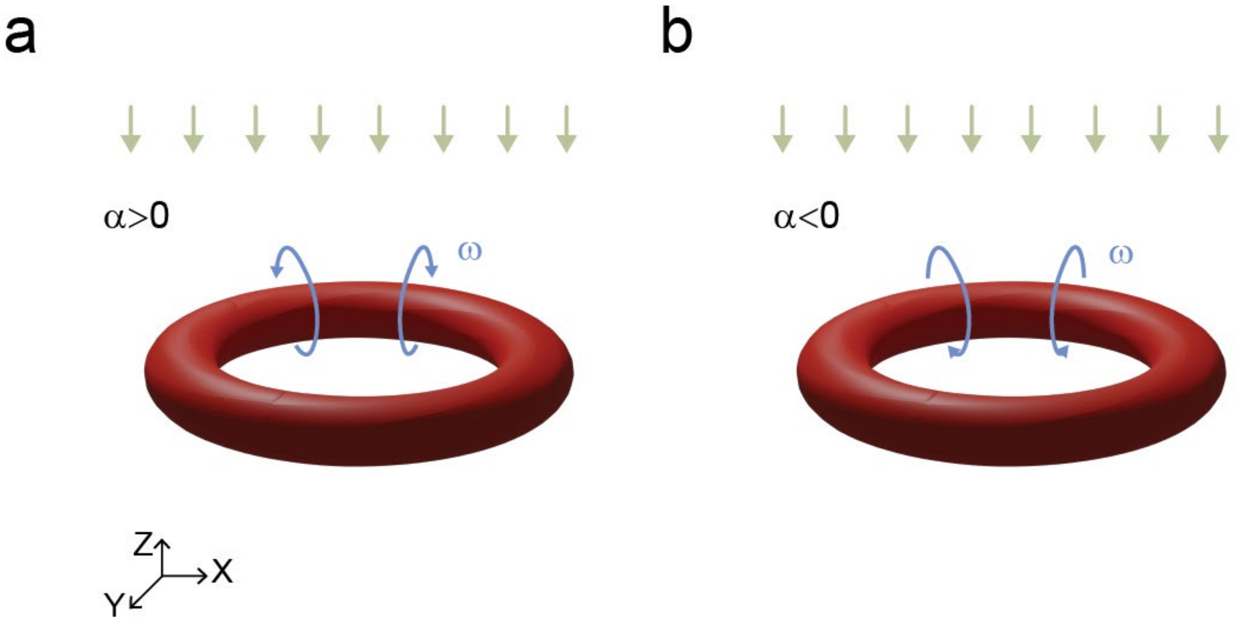

Supplementary Figure 1. Rotation classification. Schematics of light-responsive torus that (a) everts or (b) inverts under constant illumination. The eversion and inversion take place in materials with positive and negative thermal expansion coefficients (α), respectively.

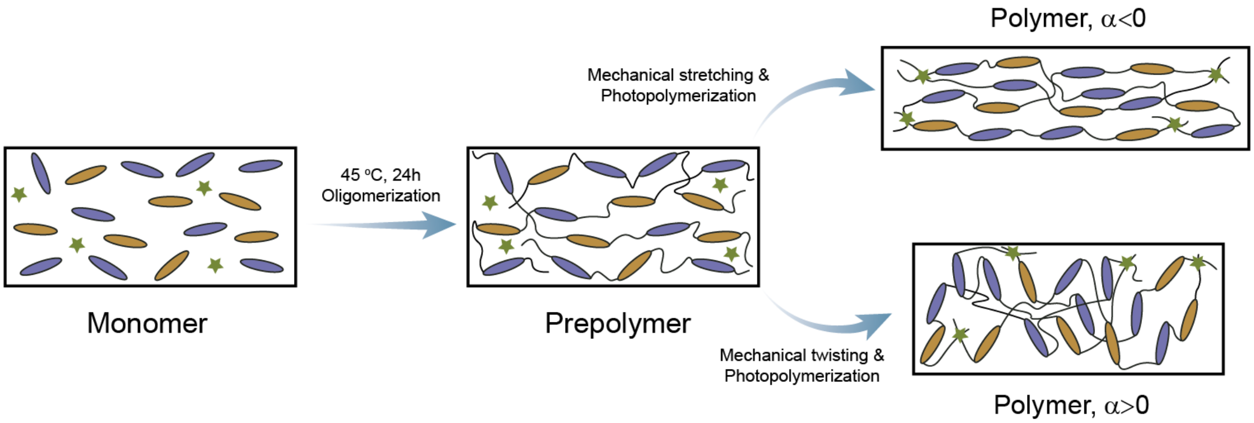

Supplementary Figure 2. Sample preparation. Synthetic steps for preparing liquid crystal elastomer fibers.

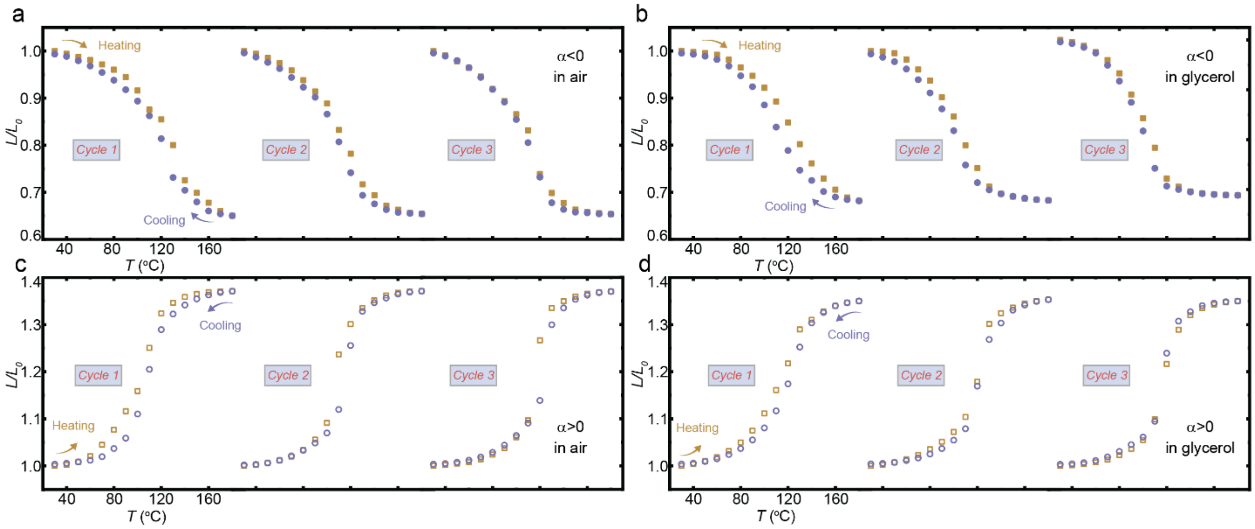

Supplementary Figure 3. Repeatable actuation test. Multiple thermal cycles for LCE fibers with (a) $\alpha < 0$ and (c) $\alpha > 0$ in air. Multiple thermal cycles for LCE fibers with (b) $\alpha < 0$ and (d) $\alpha > 0$ in glycerol.

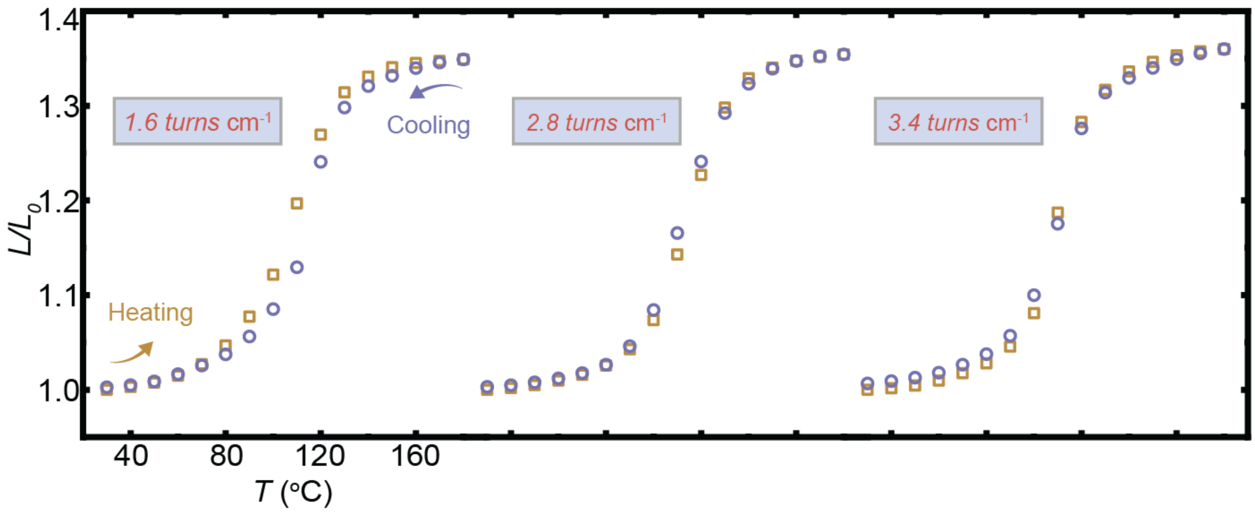

Supplementary Figure 4. The effect of twisting. Heat-induced deformation during one heating-cooling cycle for several twisting configurations.

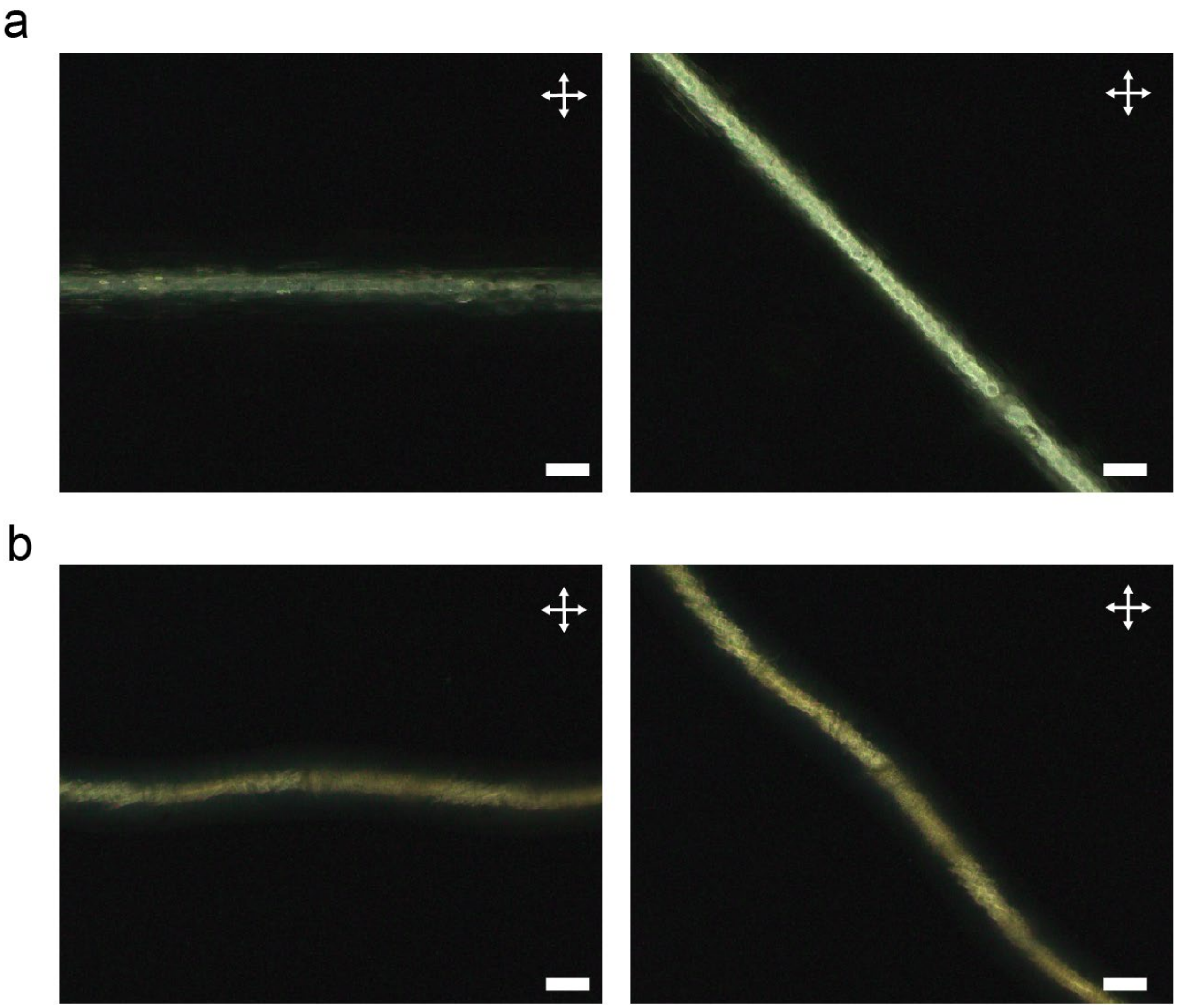

Supplementary Figure 5. Optical analysis. Polarized optical microscopy images of LCE fibers with director aligned (a) along $\alpha < 0$ and (b) perpendicular $\alpha > 0$ to the fiber axis. Scale bars: 200 μm.

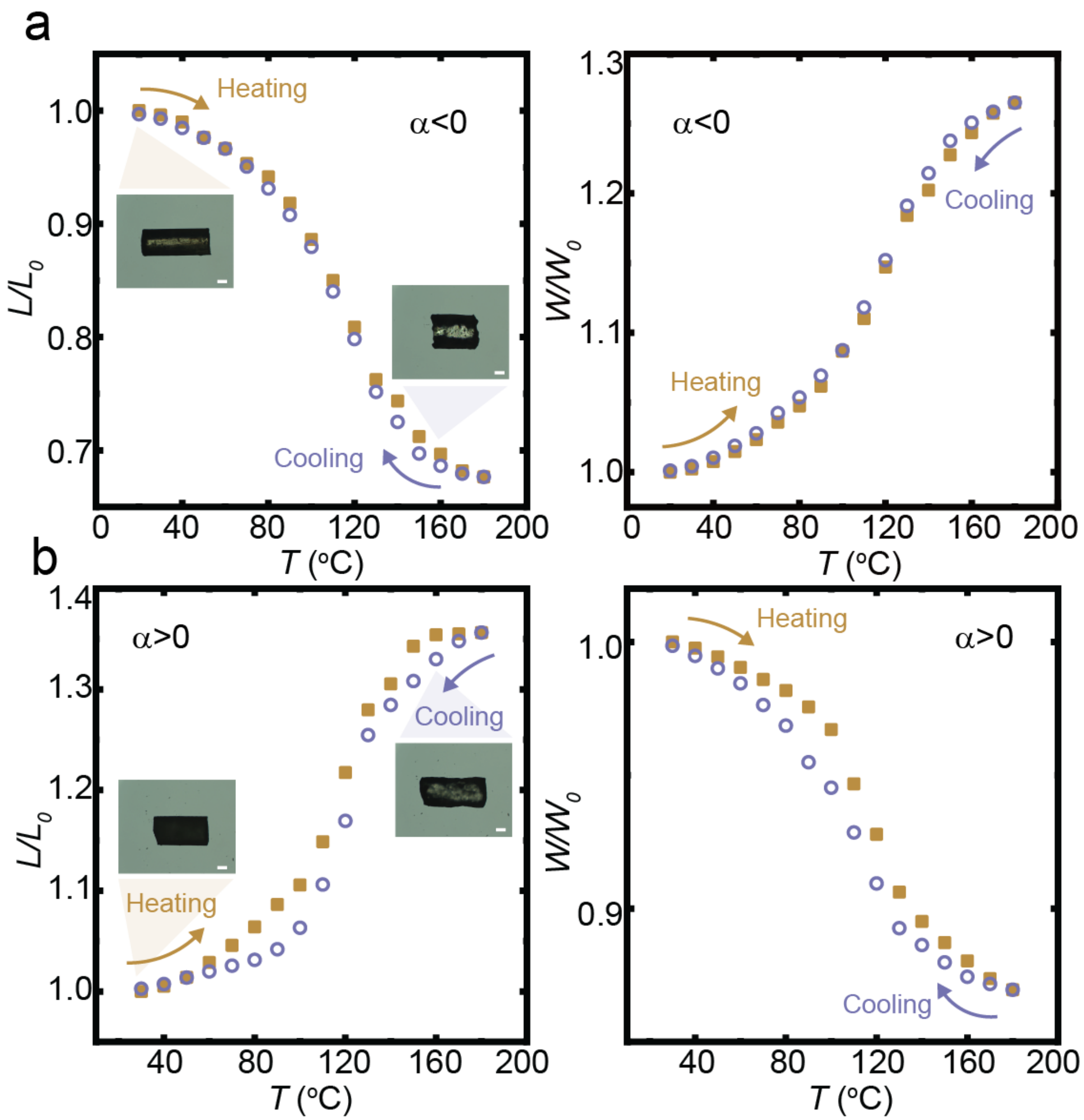

Supplementary Figure 6. Deformation capacity. Heat-induced deformation in (a) Themally contractile and (b) thermally expansive LCE fibers along (left) and perpendicular (right) to the molecular alignment direction. Insets: corresponding POM images. Scale bars: 200 μm. L_0, pristine sample length; L, length after deformation; W_0 pristine sample width; W, width after deformation.

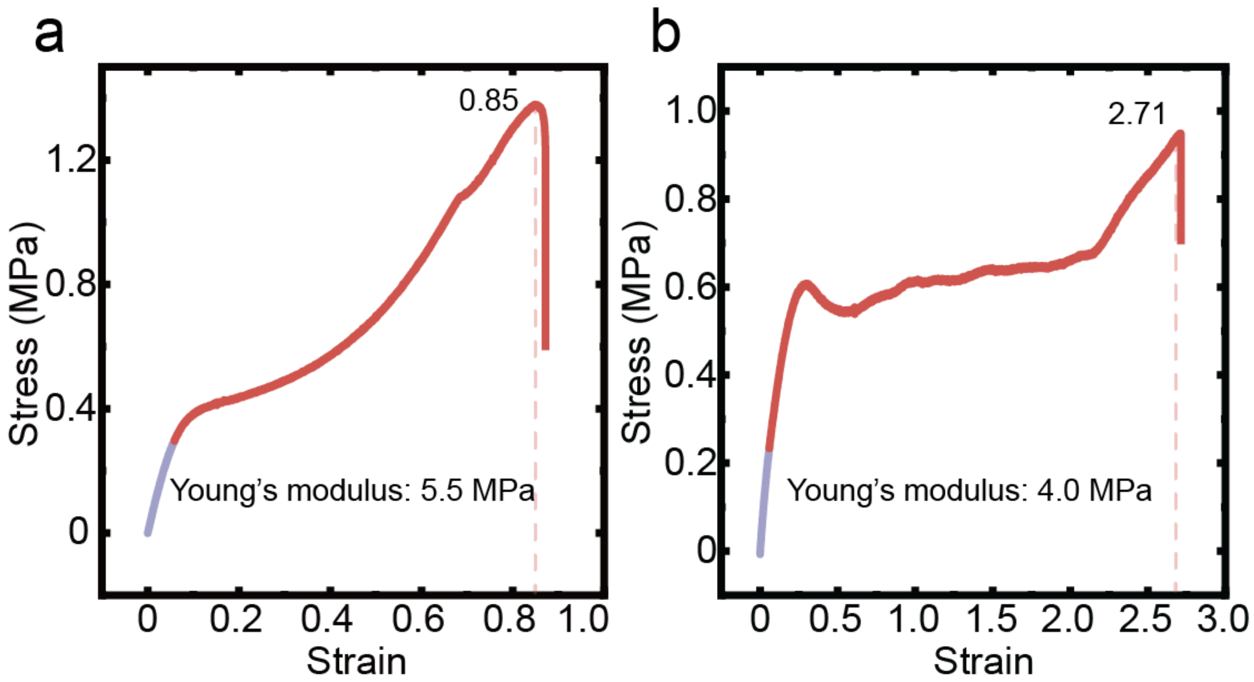

Supplementary Figure 7. Mechanical properties. Mechanical testing of LCE fibers with (a) $\alpha < 0$ and (b) $\alpha > 0$, with stretching along the long axis.

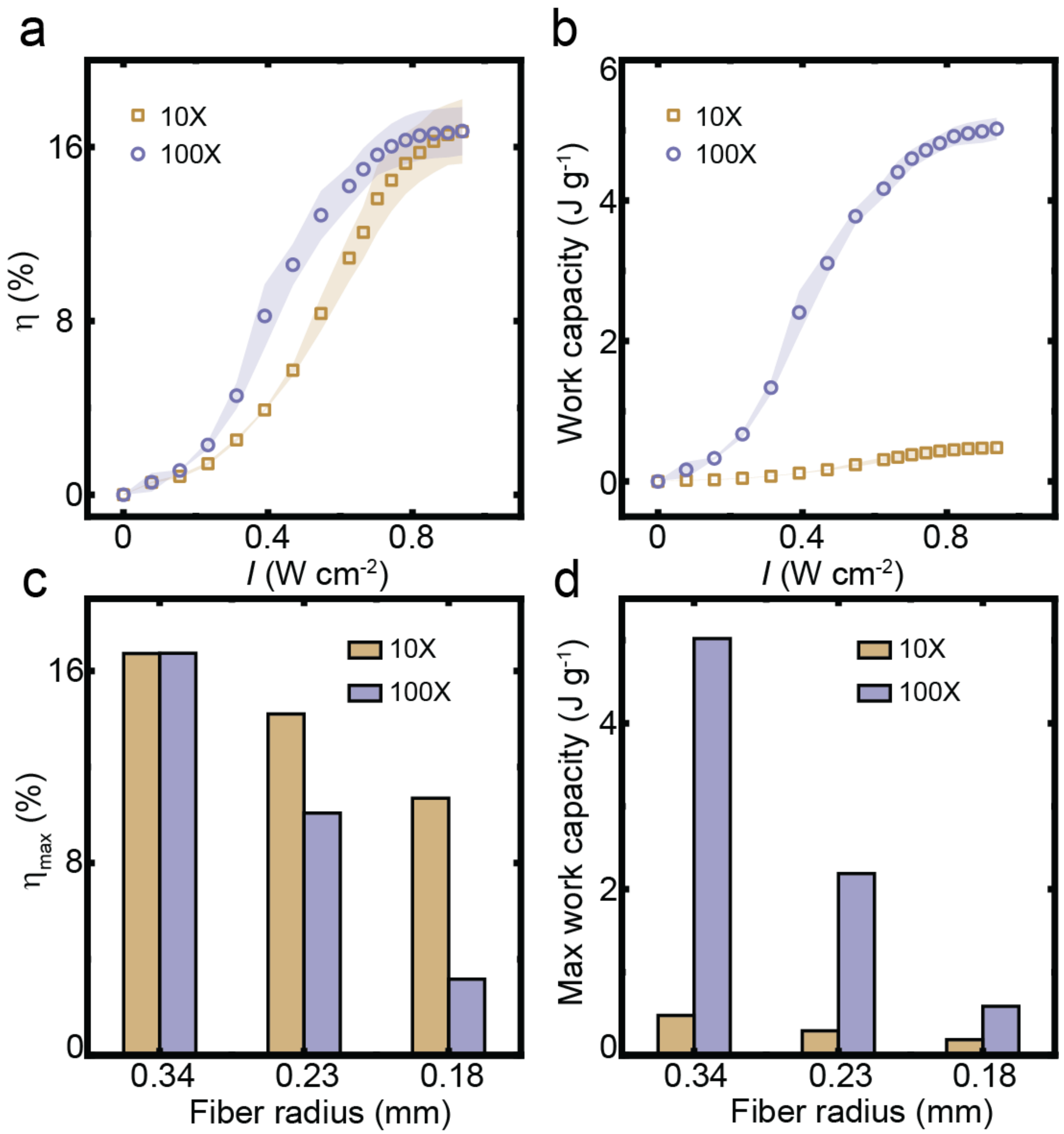

Supplementary Figure 8. Mechanical work performance. (a) Deformation strain (η) and (b) work capacity as a function of light-intensity under loading of 10X and 100X (with respect to the mass of the LCE) for an LCE with $\alpha < 0$. The effect of fiber radius on (c) maximal deformation strain and (d) work capacity under different loadings. Irradiation conditions: 532 nm, 0.9 W cm^{-2}. The error bars in (a), (b) are displayed as mean values +/- standard deviation (n = 3). The same sample was measured repeatedly.

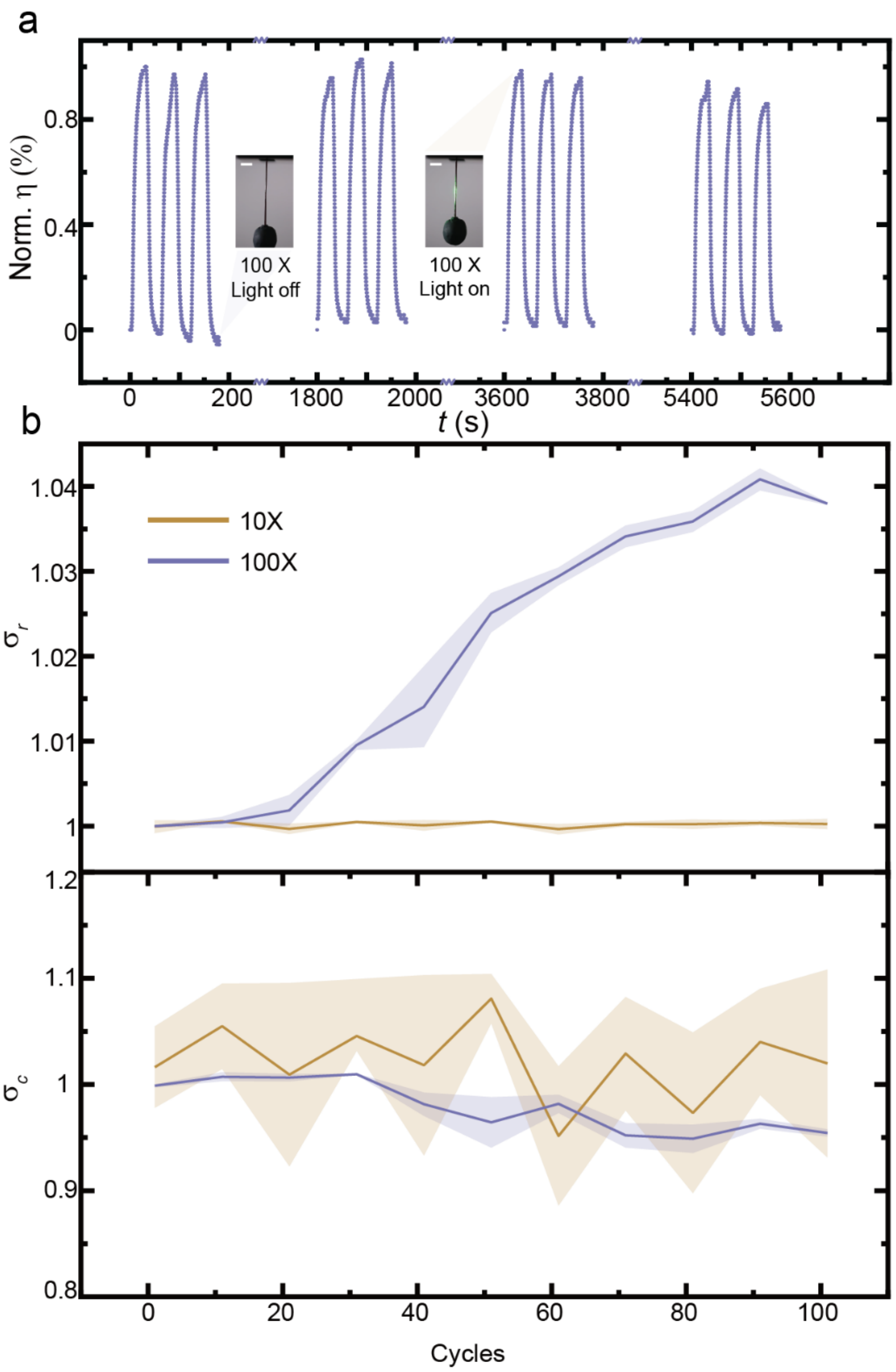

Supplementary Figure 9. Reversibility of the light-induced deformation. (a) Deformation strain over hundred light actuation cycles under 100X (with respect to the mass of the LCE) loading. Insets: photographs of the light-induced deformation. Scale bars: 5 mm. (b) Deformation reversibility (σ_c) and shape recovery (σ_r) over hundred light actuation cycles under 10X and 100X loadings. Irradiation conditions: 532 nm, 0.4 W cm^{-2}. The error bars in (b) are displayed as mean values +/- standard deviation (n = 3). The same sample was measured repeatedly.

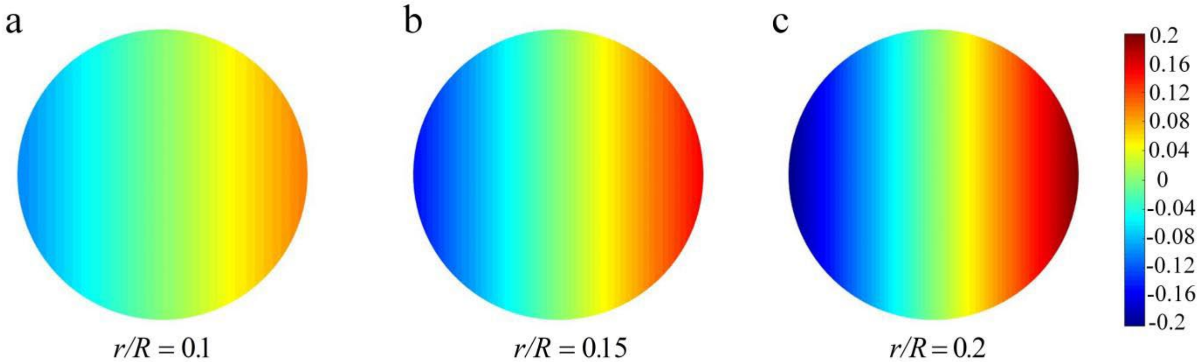

Supplementary Figure 10. Calculation of the static strain field. (a) $\frac{r}{R} = 0.1$; (b) $\frac{r}{R} = 0.15$; (c) $\frac{r}{R} = 0.2$. With increasing slenderness, the static strain field increases, as shown in Eq. (2).

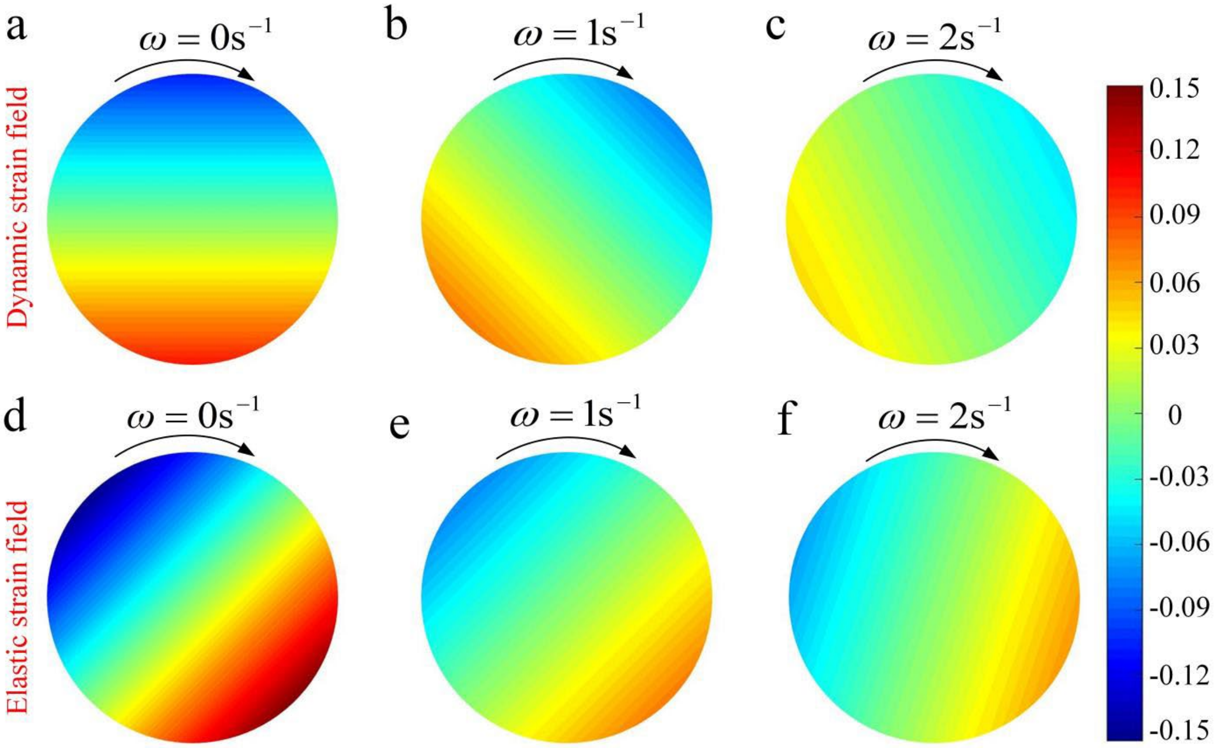

Supplementary Figure 11. Numerical calculation of dynamic strain field and elastic strain field. (a) and (d) $\omega = 0$ s^{-1}; (b) and (e) $\omega = 1$ s^{-1}; (c) and (f) $\omega = 2$ s^{-1}. Other parameters are set to be $\tau = 1$ s, $p = 0.5$ K s^{-1}, $\alpha =$ -0.002 K^{-1}, and $\frac{r}{R} = 0.1$. With the increase of angular frequency, the dynamic strain field decreases and exhibits asymmetry. Total elastic strain field compromises static and dynamic strain fields. Details see in Eqs. (8) and (7).

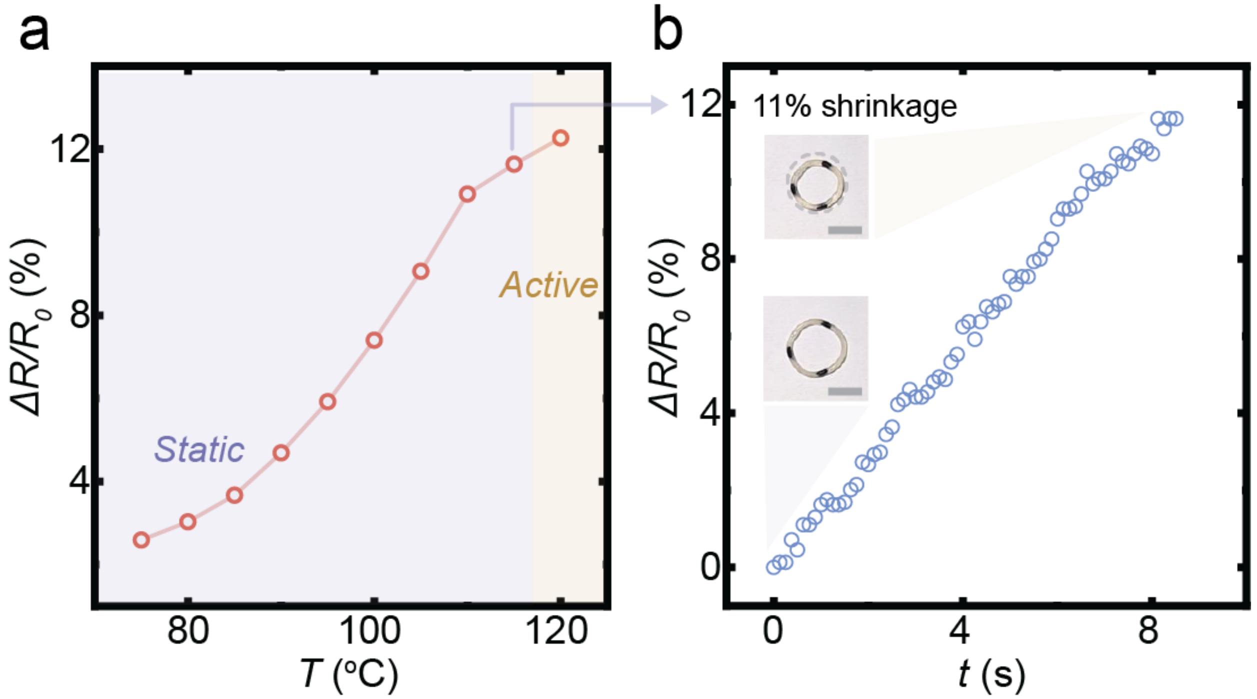

Supplementary Figure 12. Thermally driven dimension shrinkage. (a) Torus size decrease ($\alpha < 0$) under different temperatures. (b) Shrinkage dynamics on a 115 °C hot plate. Insets: photographs of the static torus before (bottom) and after (top) shrinking. Scale bars: 2 mm.

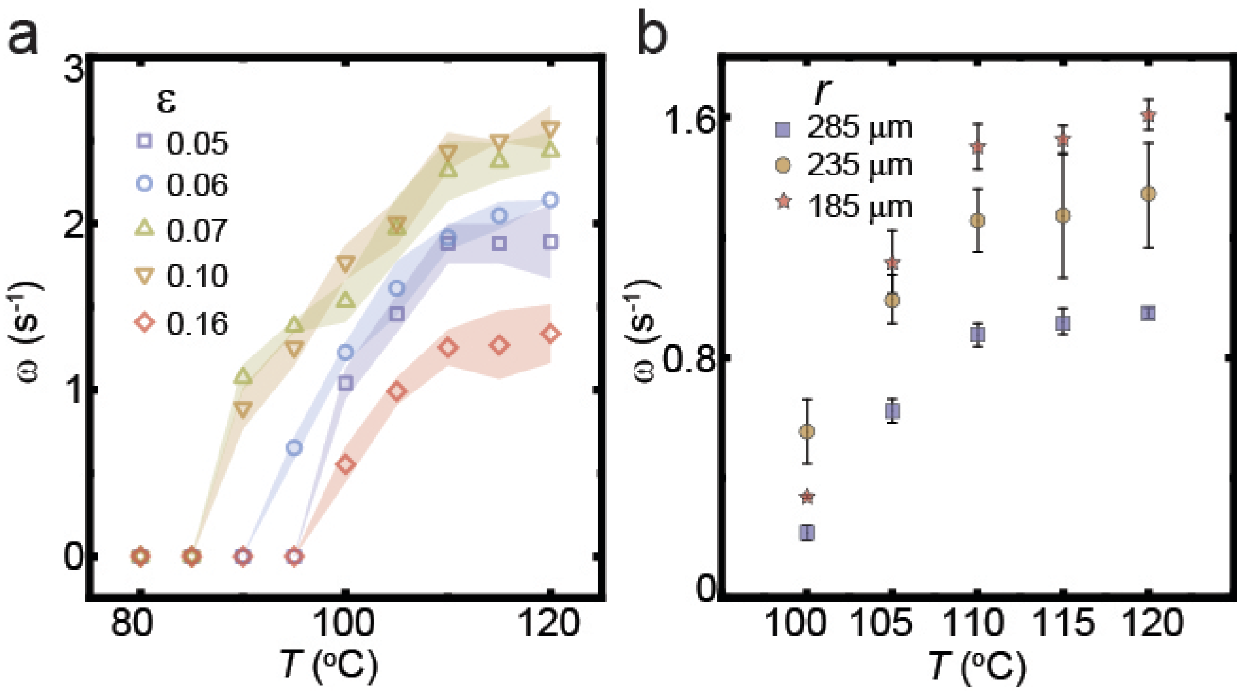

Supplementary Figure 13. Thermally driven eversion. Torus (a) slenderness and (b) fiber radius affect the angular frequency of rotation (ω) obtained at different temperatures. The error bars are displayed as mean values +/- standard deviation (n = 3). The same sample was measured repeatedly.

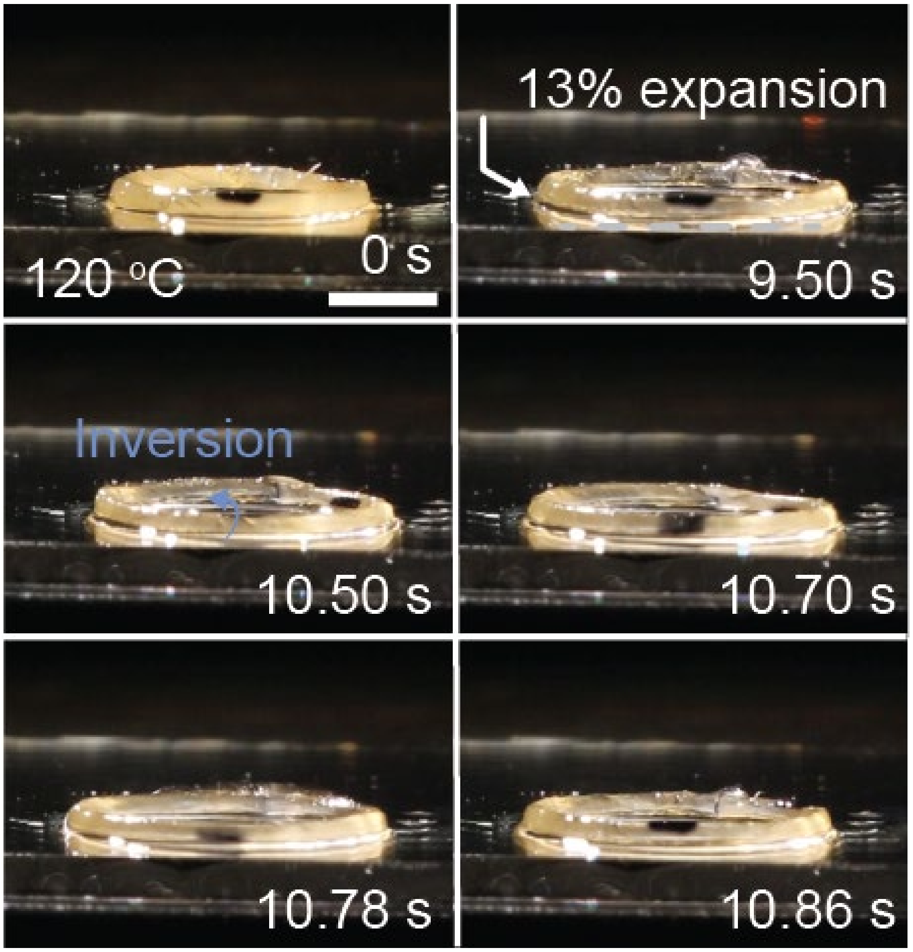

Supplementary Figure 14. Thermally driven inversion. Photographs of active torus with positive thermal expansion coefficient ($\alpha > 0$) rotating on a hot plate. Scale bar: 2 mm.

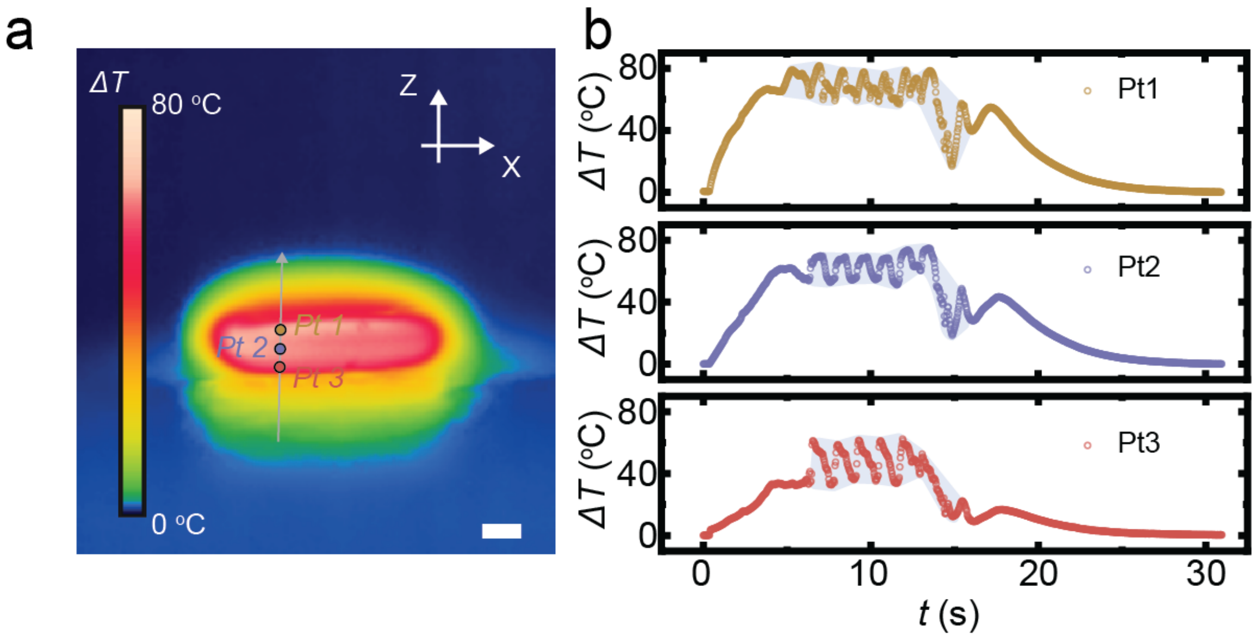

Supplementary Figure 15. Temperature oscillation of the active torus. (a) Infrared image of the rotational torus at t = 9.3 s. Along the Z direction, the temperature evolution at points 1, 2 and 3 are depicted in (b). Irradiation conditions: 532 nm, 1.8 W cm^{-2}. Scale bar: 1 mm.

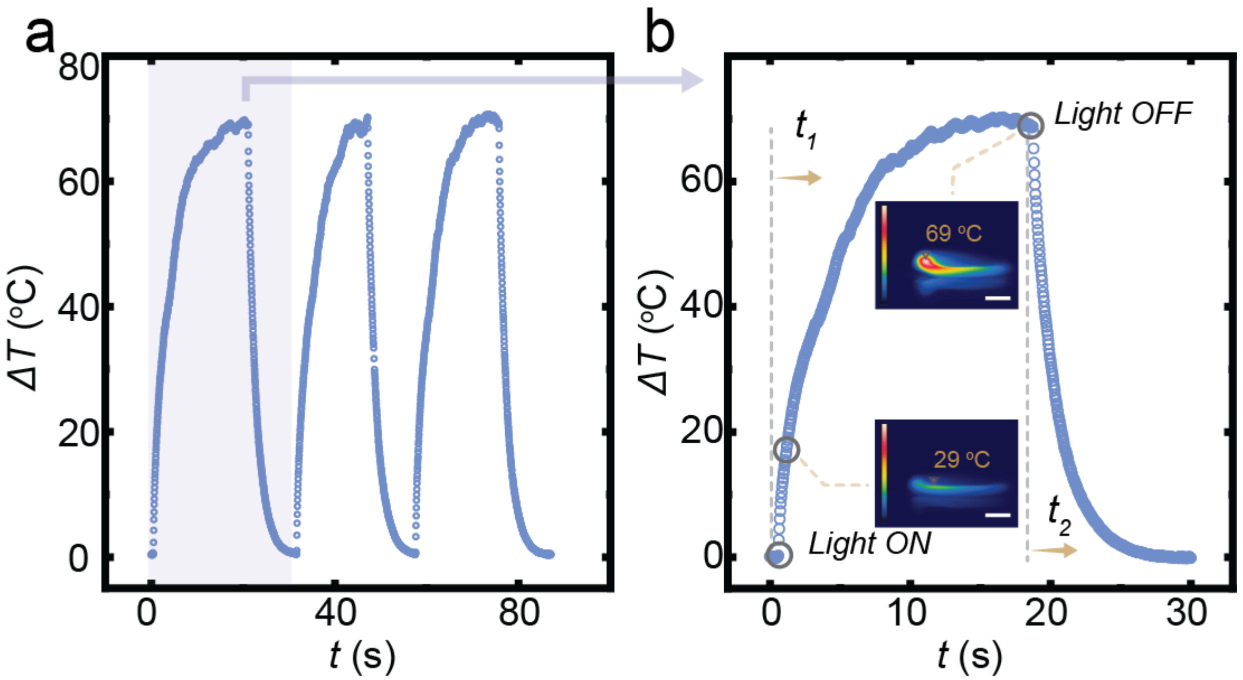

Supplementary Figure 16. Photothermal heating kinetics. (a) Temporal evolution of the temperature difference ΔT. (b) ΔT under light illumination. t_1 represents the duration from the pristine state to the maximal ΔT state. t_2 represents the duration from the maximal ΔT state to the pristine state after ceasing the light illumination. Irradiation conditions: 532 nm, 1.3 W cm^{-2}. Insets: infrared images of the torus in the different stages of a rotation cycle. Scale bars: 1 mm.

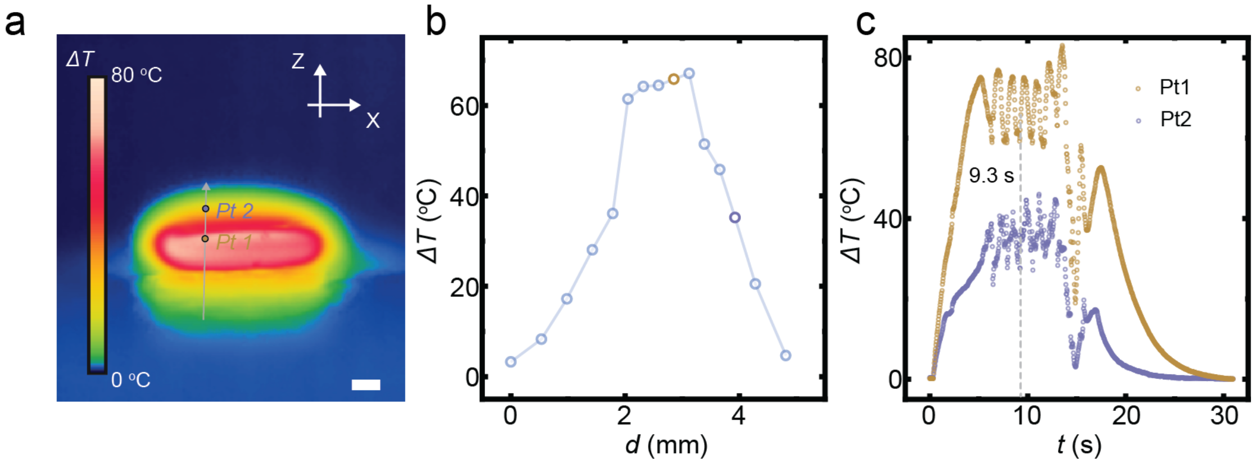

Supplementary Figure 17. Photothermal heating gradient. (a) Infrared photograph of the rotational torus at $t = 9.3$ s. Along the Z direction, points 1 and 2 are marked with different colors. (b) Temperature difference ΔT along the measured line at $t = 9.3$ s. (c) Temporal evolution of ΔT over a few rotation cycles, measured at points 1 and 2. Irradiation conditions: 532 nm, 1.8 W cm^{-2}. Scale bar: 1 mm.

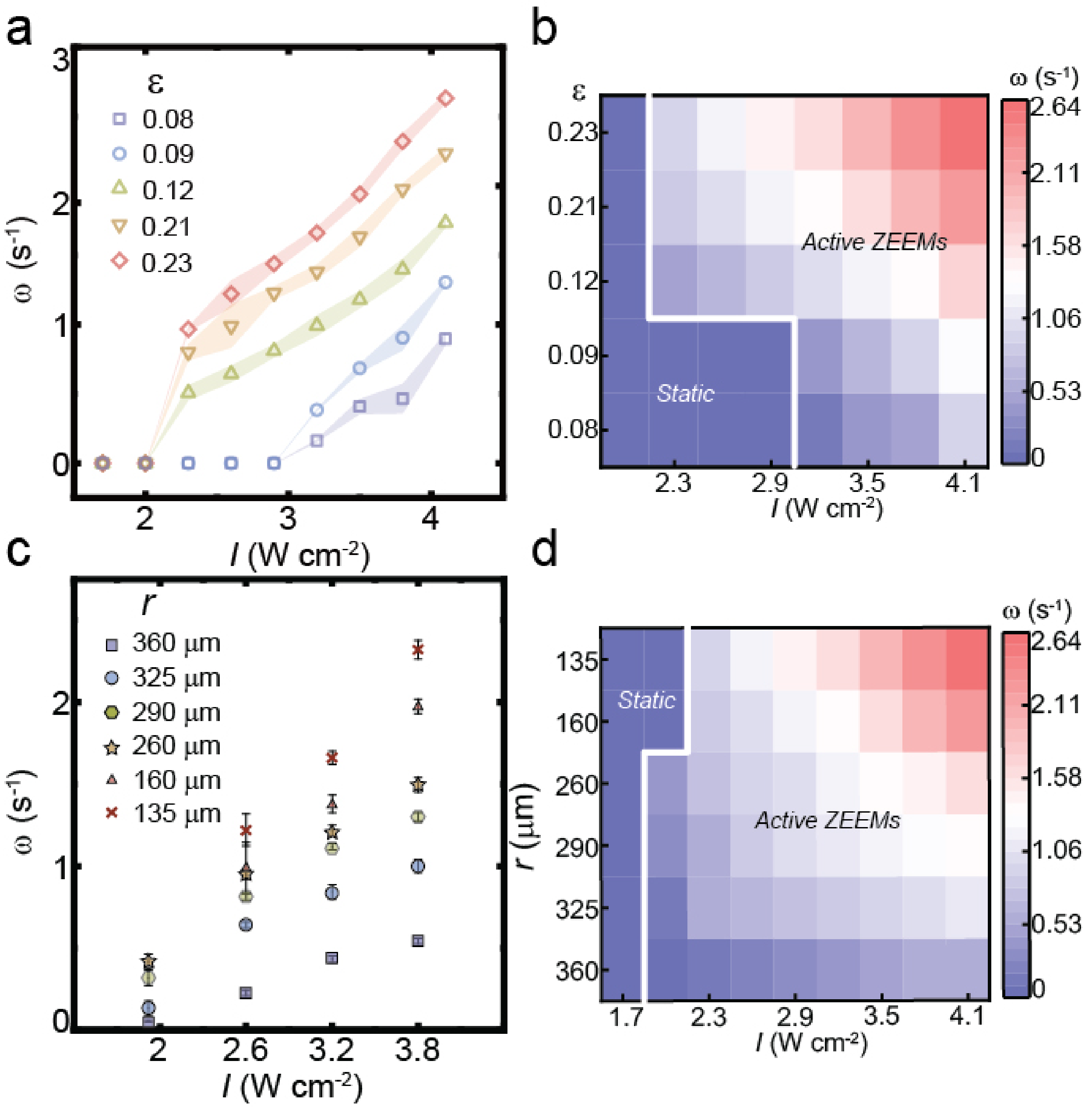

Supplementary Figure 18. Optically driven inversion. Torus slenderness (a) and fiber radius (c) affect the angular frequency ω of rotation on glycerol surface. The corresponding color map summarizations are given in (b) and (d). Irradiation conditions: 532 nm, different light intensities. The error bars in (a), (c) are displayed as mean values +/- standard deviation (n = 3). Each data point in (b), (d) is presented as the mean value derived from three measured values. The same sample was measured repeatedly.

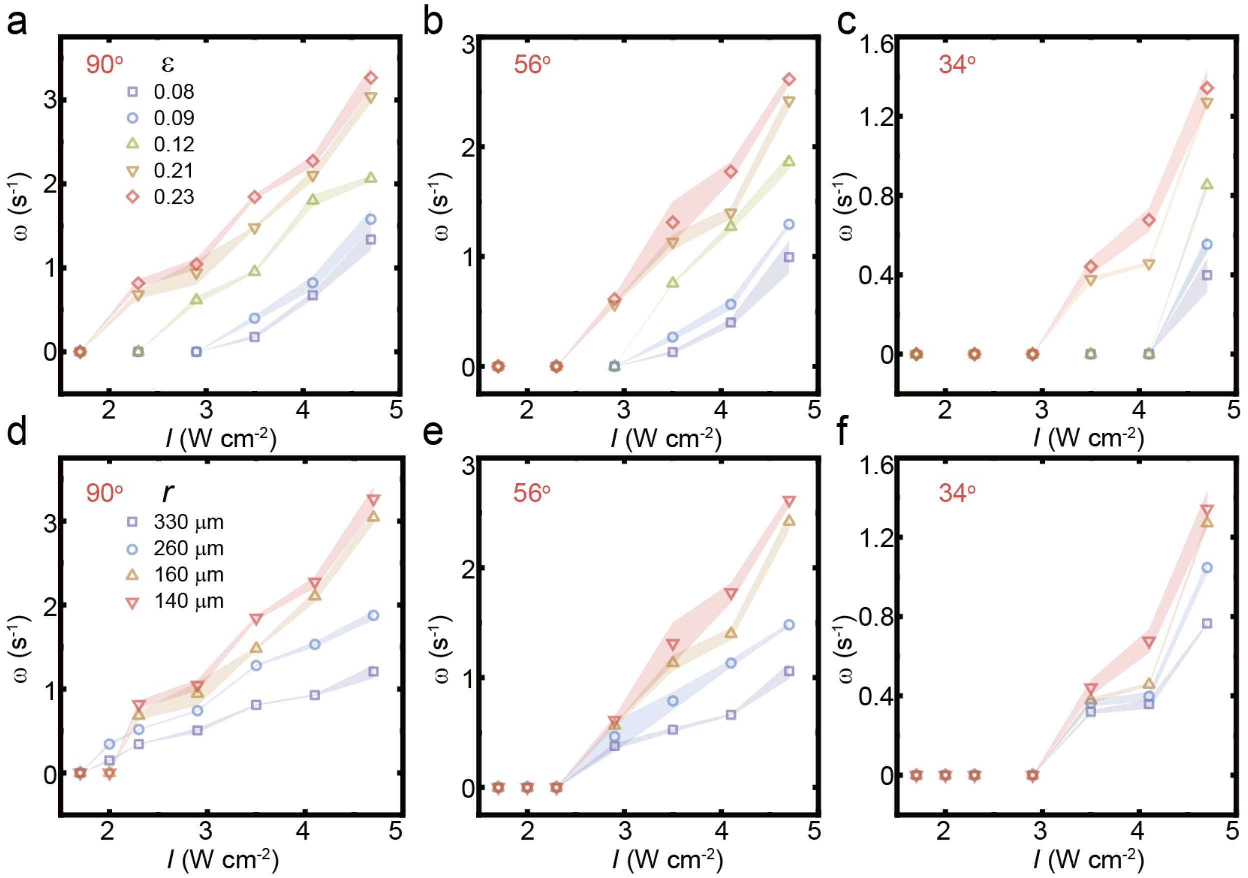

Supplementary Figure 19. Rotation under oblique illumination. Torus slenderness (a-c) and fiber radius (d-f) affect the angular frequency ω of rotation on glycerol surface when the torus is illuminated from different angles. Irradiation conditions: 532 nm, different light intensities. The error bars are displayed as mean values +/- standard deviation (n = 3). The same sample was measured repeatedly.

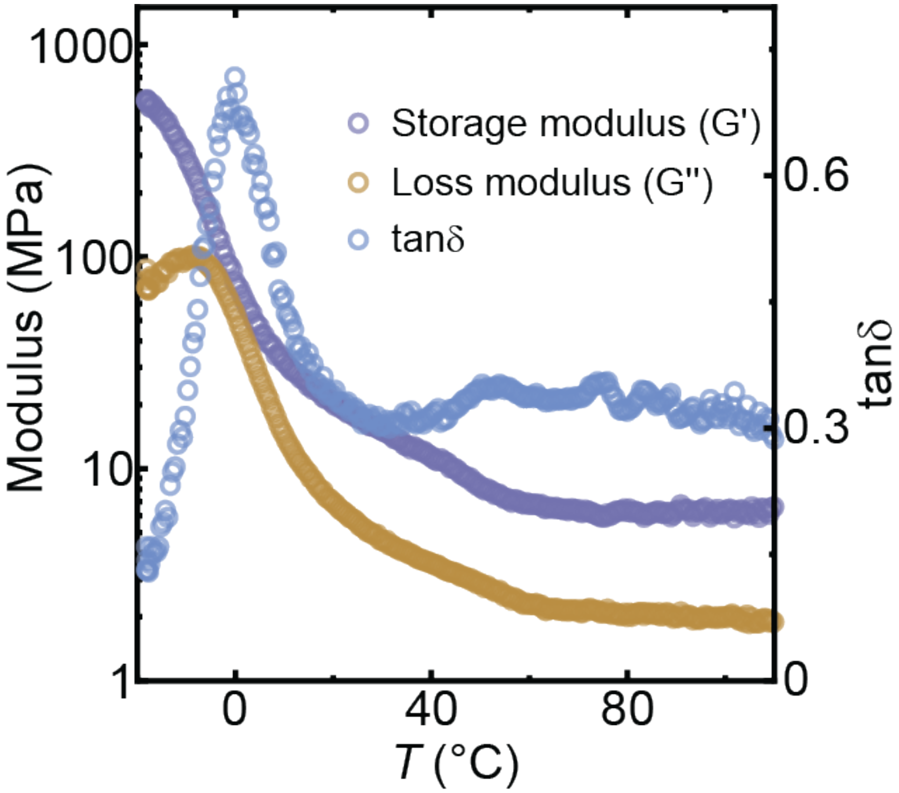

Supplementary Figure 20. Mechanical testing. Temperature effect on mechanical properties scanned at 1Hz.

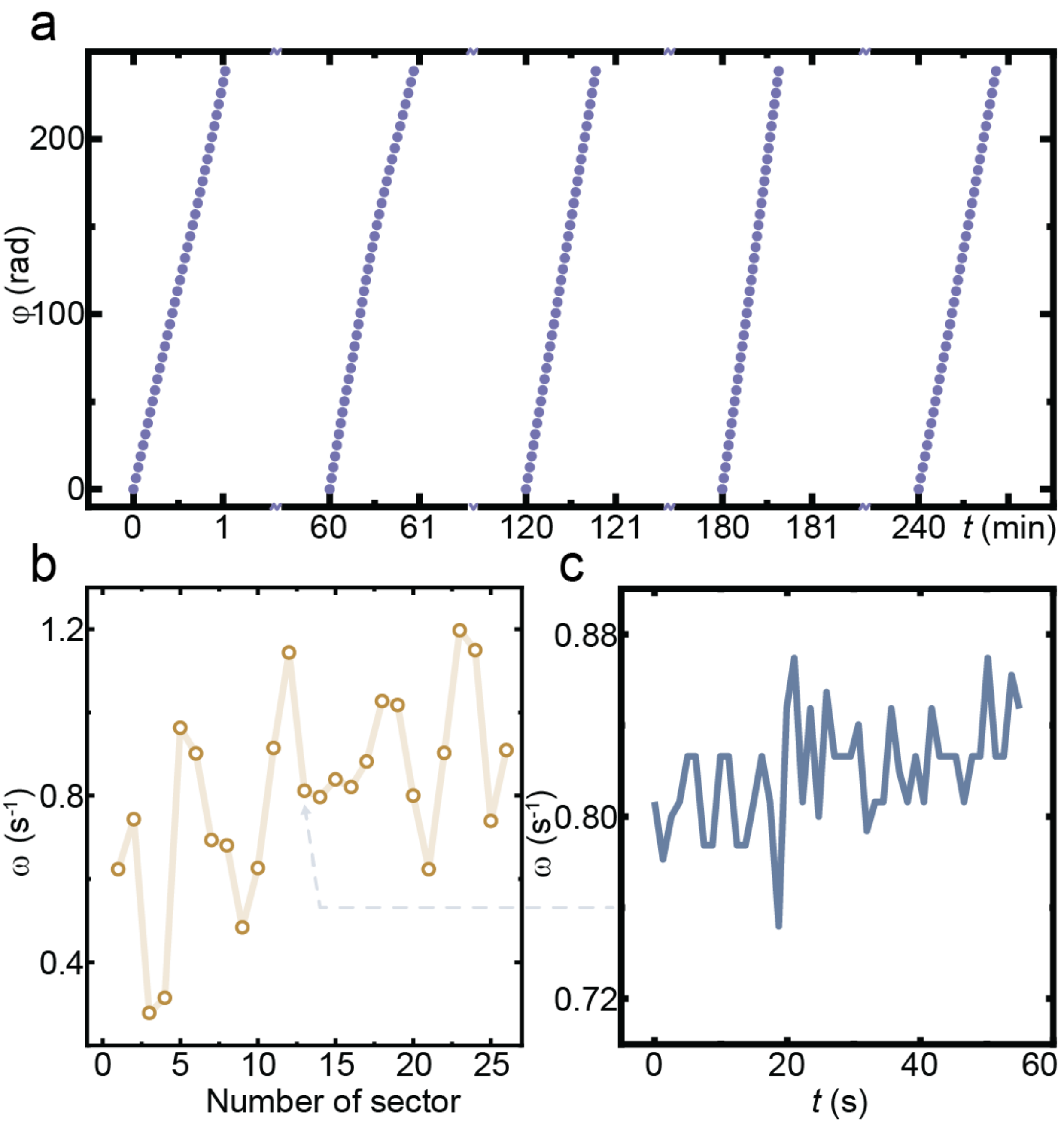

Supplementary Figure 21. Rotation stability of the torus. (a) Cumulative rotational angle during the first minute of each hour during 4 hours of continuous rotation. (b) Average angular frequency in the first minute of every 10 min interval. (c) Real-time angular frequency of rotation in the thirteenth sector. Irradiation conditions: 532 nm, 2.9 W cm^{-2}.

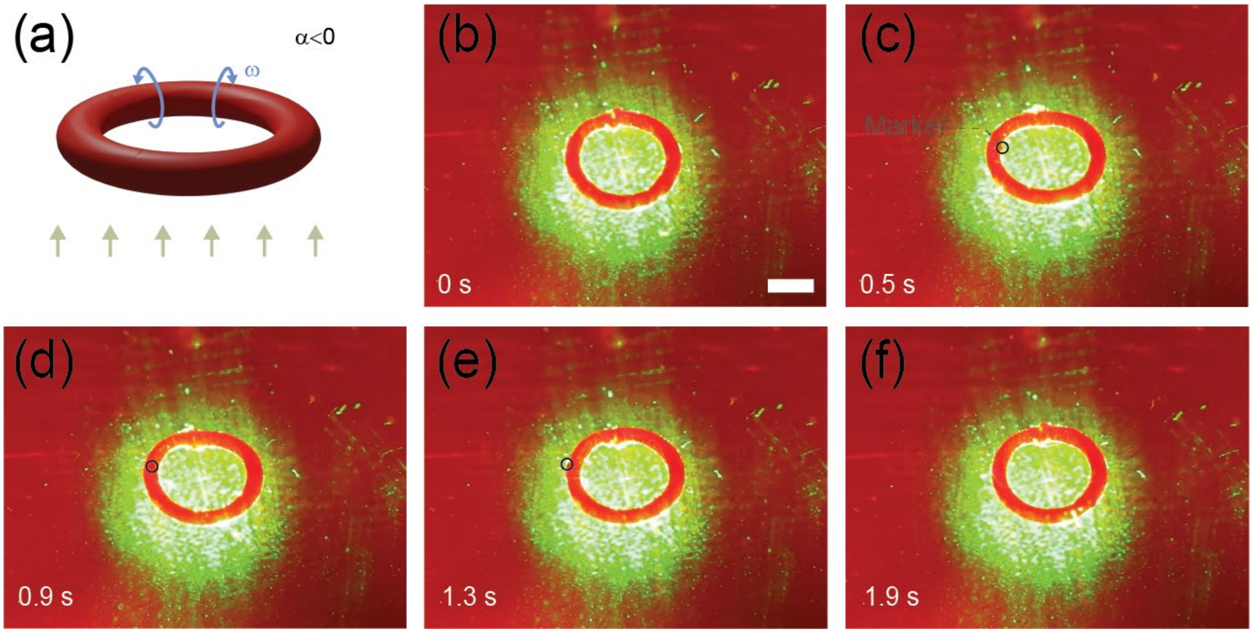

Supplementary Figure 22. Illumination from bottom. (a) Schematics, and (b) photographs of active torus with $\alpha < 0$ everting when illuminated from the bottom. Irradiation conditions: 532 nm 3.2 W cm^{-2}. Scale bar: 2 mm.

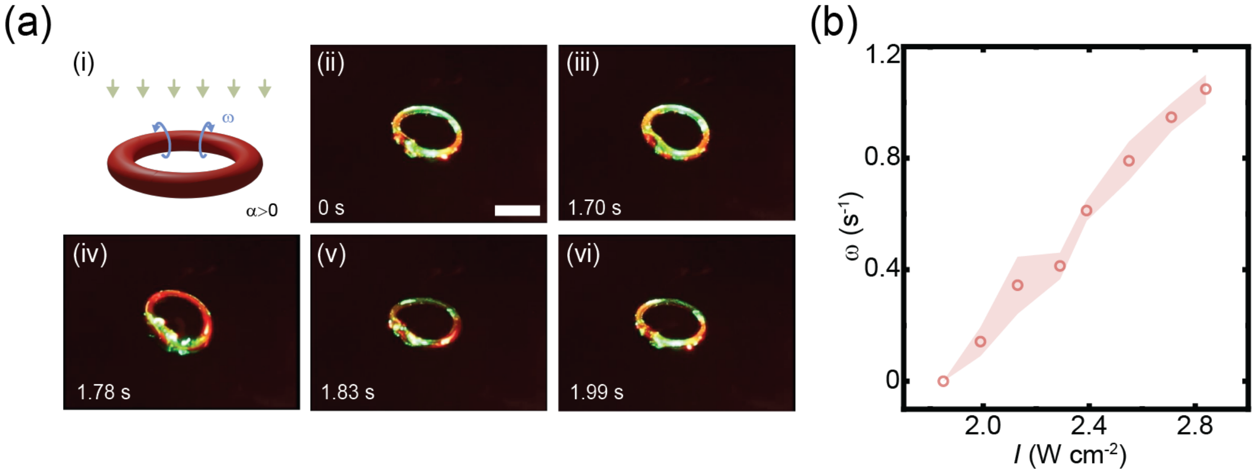

Supplementary Figure 23. Optically expansive everting rotation. (a) i, Schematics of a torus with $\alpha > 0$ everting under illumination from the top, and ii-vi, corresponding photographs. Irradiation conditions: 532 nm, 2.0 W cm^{-2}. Scale bar: 2 mm. (b) Corresponding plot of angular frequency as a function of light intensity. The error bars are displayed as mean values +/- standard deviation (n = 3). The same sample was measured repeatedly.

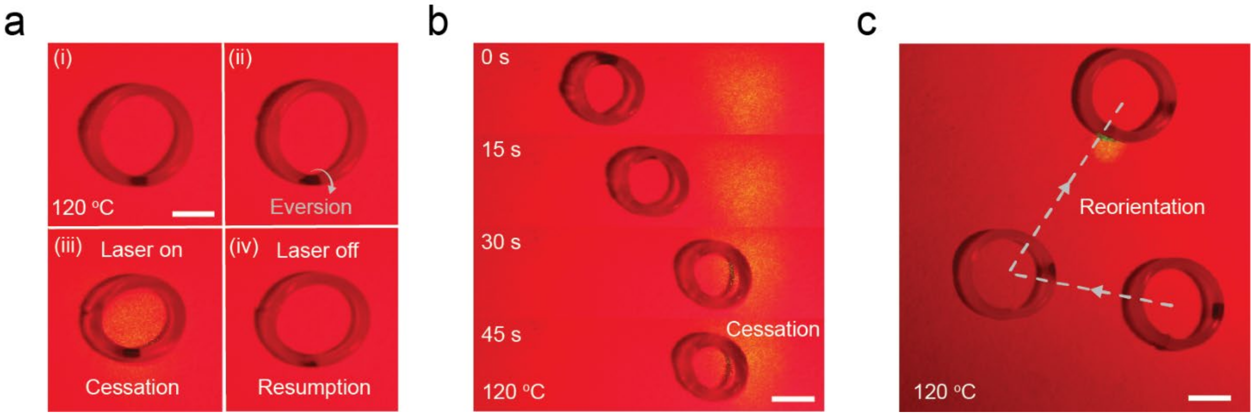

Supplementary Figure 24. Perception to the environmental light field. (a) Photographs of an everting torus on a hot plate ceasing and resuming its rotation in response to environmental light field. (b) Translational motion of the torus on a hot plate can be ceased when exposed to a laser beam, and (c) the translation direction can be steered with oblique illumination. Irradiation conditions: 532 nm, 0.2 W cm^{-2}. Scale bars: 2 mm.

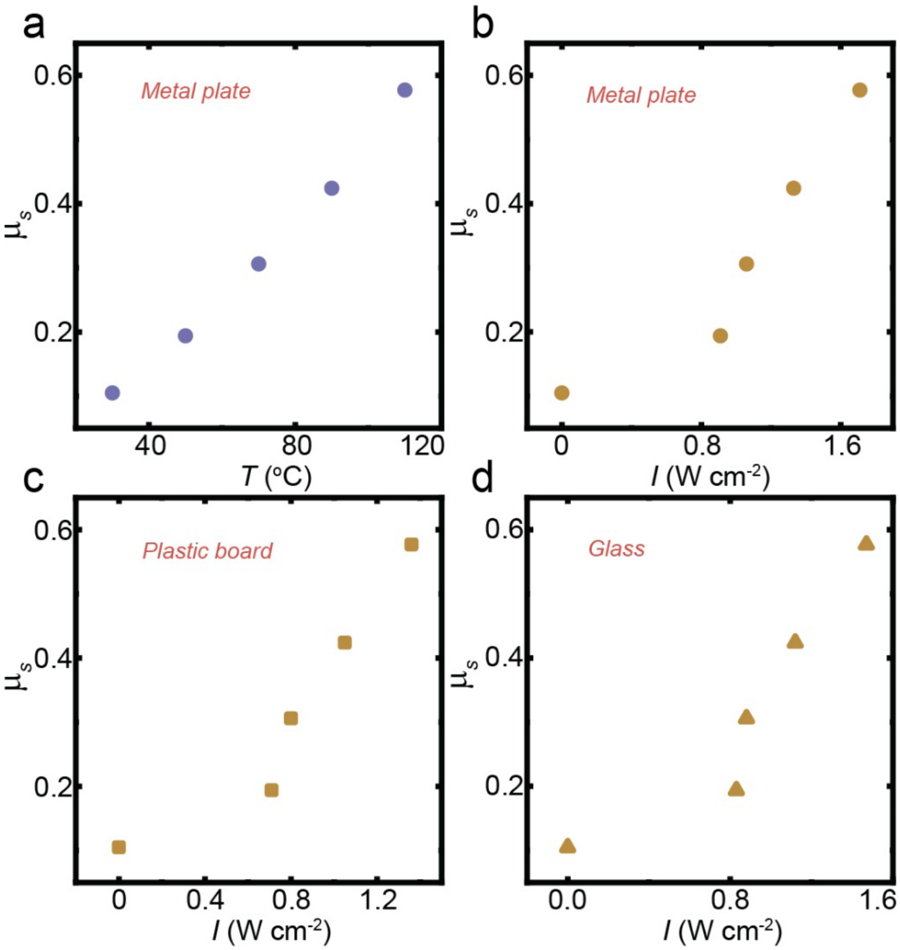

Supplementary Figure 25. Temperature-dependent friction on different surfaces. Static friction coefficient μ_s, measured via an inclined plane method, as a function of (a) temperature, on a metal plate. μ_s as a function of light intensity (532 nm) on a (b) metal plate, (c) acrylic board and (d) glass substrate. Metal plate: Thorlabs (BA2/M). Plastic board: Petri dishes, polystyrene. Glass substrate: microscope slides (Epredia).

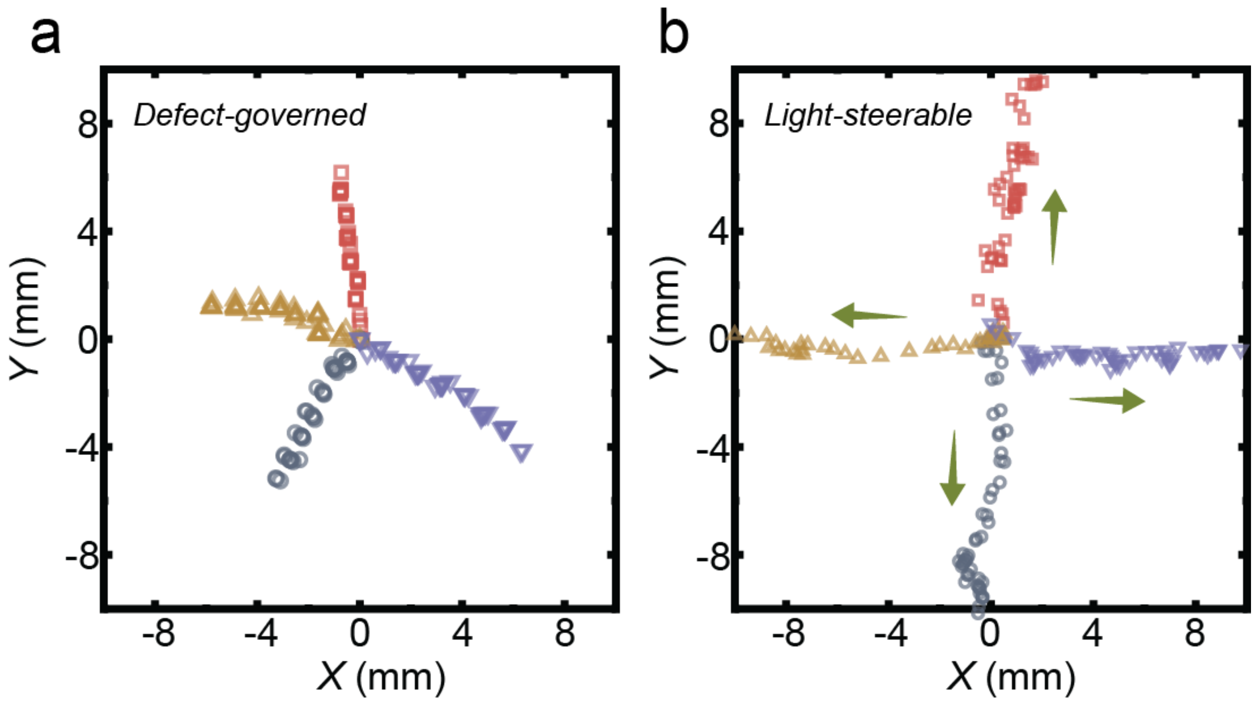

Supplementary Figure 26. Locomotion comparison. (a) Trajectories of same sample locomoting stochastically on a hot plate at 120 °C by simply placing the defective sample differently. Each color represents one course in a time span of 20 s. (b) Trajectories of same sample under oblique illumination for a time span of 20 s. Green arrows represent oblique incidence direction, where the LCE walks away from the light source. Irradiation conditions: 532 nm, 1.9 W cm^{-2}.

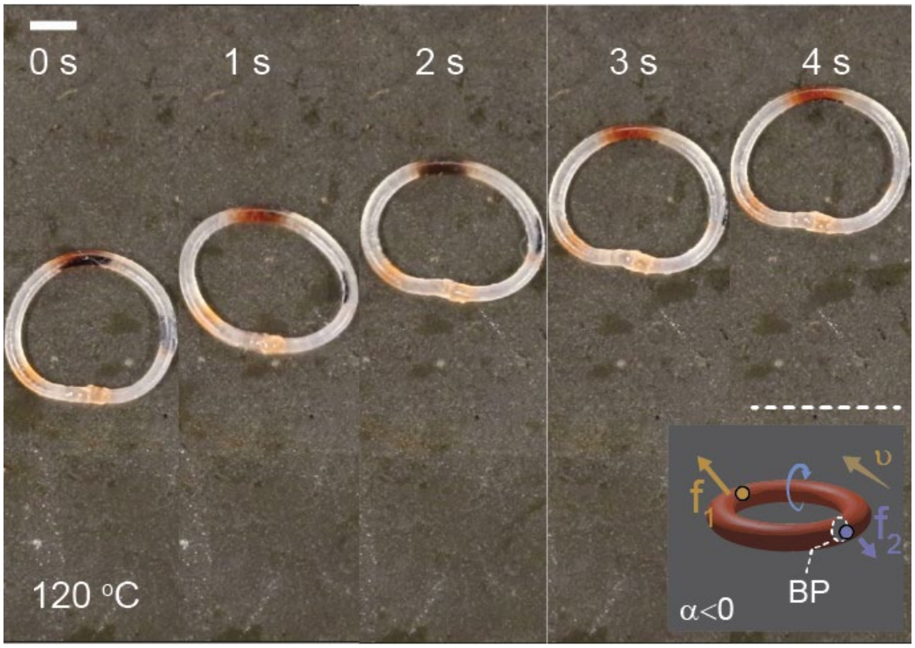

Supplementary Figure 27. Defect-determined motion. Photographs of the active torus with amplified binding point (BP) locomoting on a hot plate. Inset: corresponding force analysis. Scale bar: 2 mm.

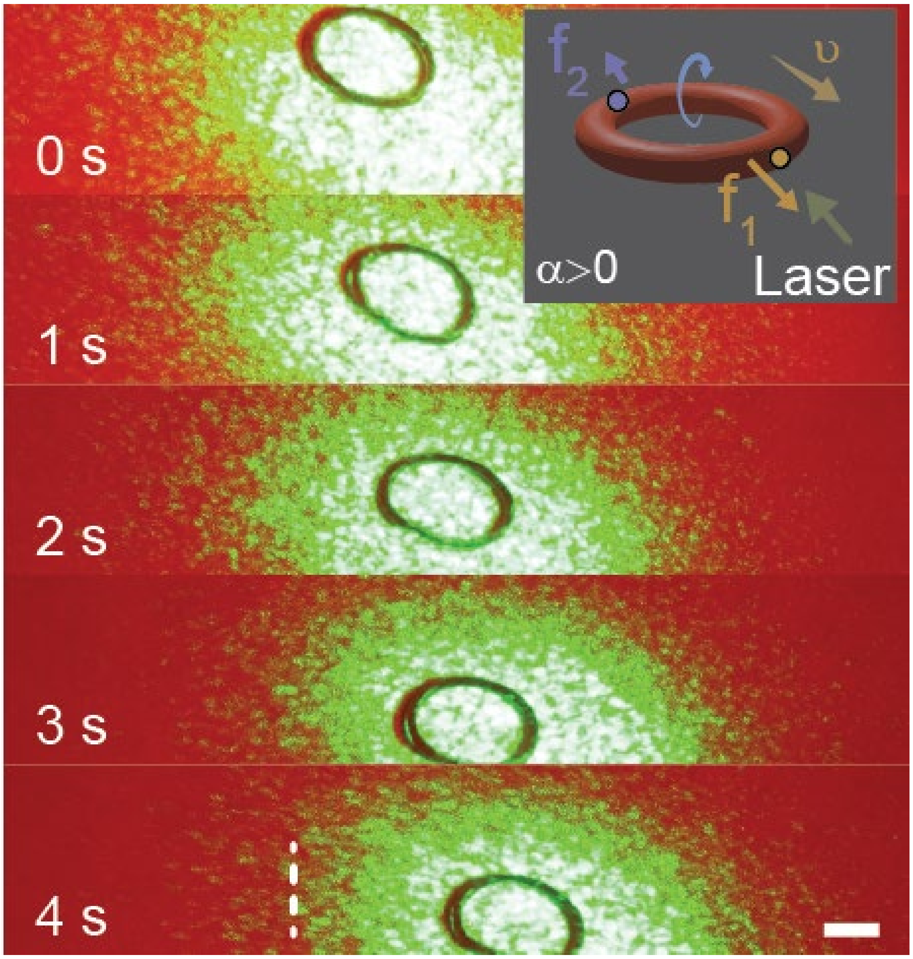

Supplementary Figure 28. Terrestrial locomotion in air for LCE with $\alpha > 0$. Photographs of the active torus locomoting on dry land towards the light source under oblique illumination. Inset: corresponding forces analysis. Irradiation conditions: 532 nm, 1.9 W cm^{-2}. Scale bar: 2 mm.

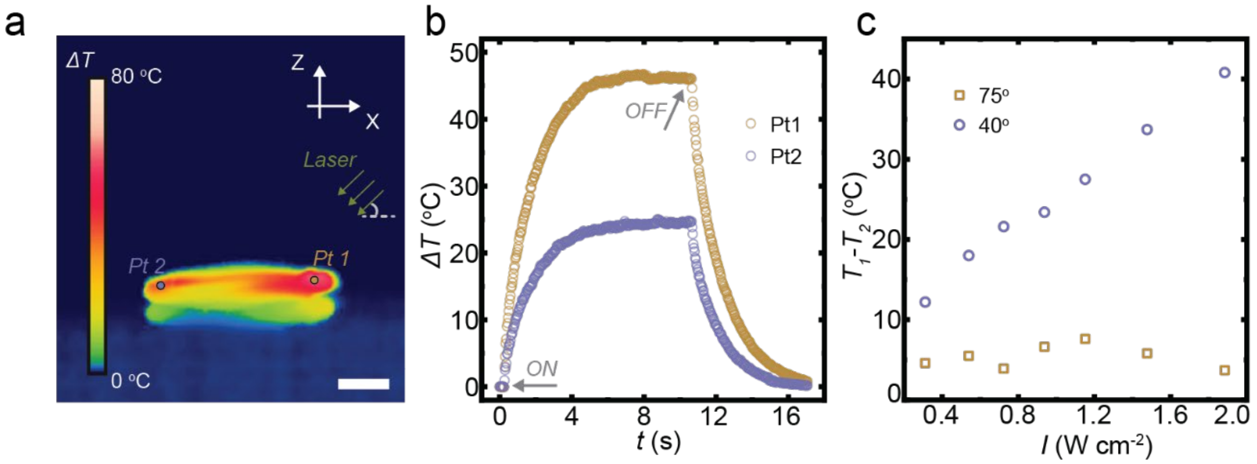

Supplementary Figure 29. Temperature difference under oblique illumination. (a) Infrared image of the torus under oblique illumination. Irradiation conditions: 532 nm, 0.7 W cm^{-2}. Scale bar: 1 mm. (b) Temporal evolution of ΔT measured at points 1 and 2 by switching ON-OFF the irradiation. (c) Influence of inclination angle on the temperature difference at point 1 and 2 under different light intensities.

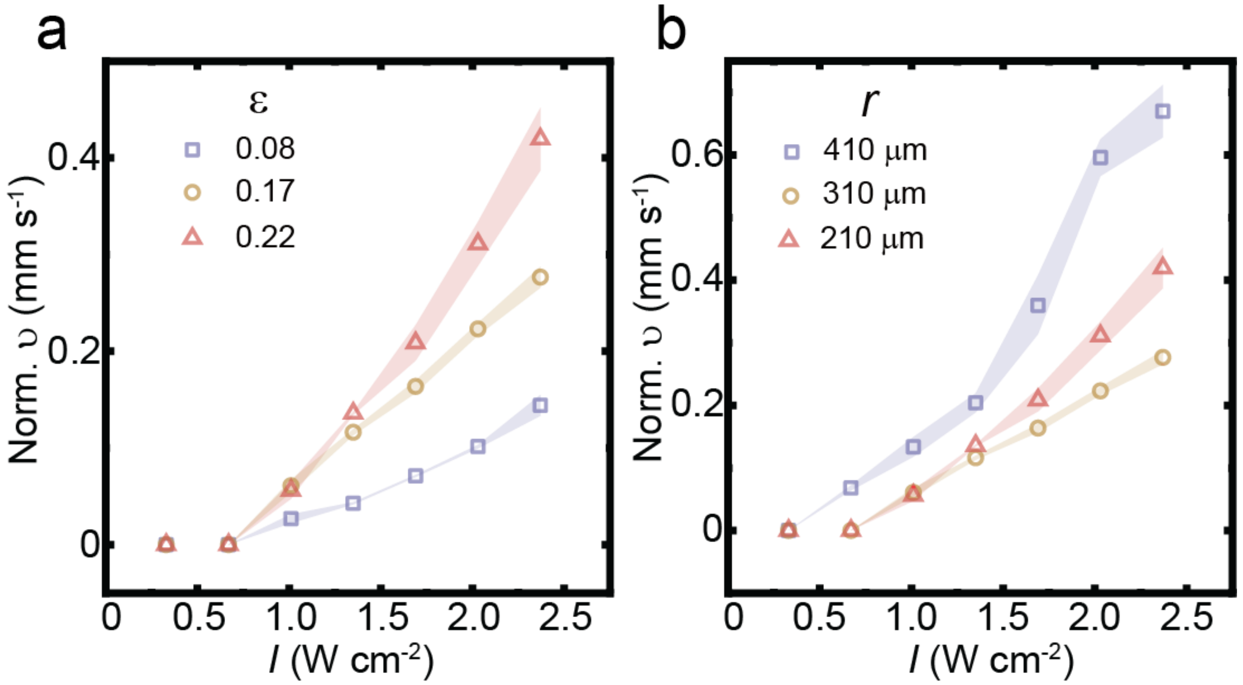

Supplementary Figure 30. Terrestrial locomotion. Torus slenderness (a) and fiber radius (b) affect the normalized translational velocity υ. Irradiation conditions: 532 nm, different light intensities. The error bars are displayed as mean values +/- standard deviation (n = 3). The same sample was measured repeatedly.

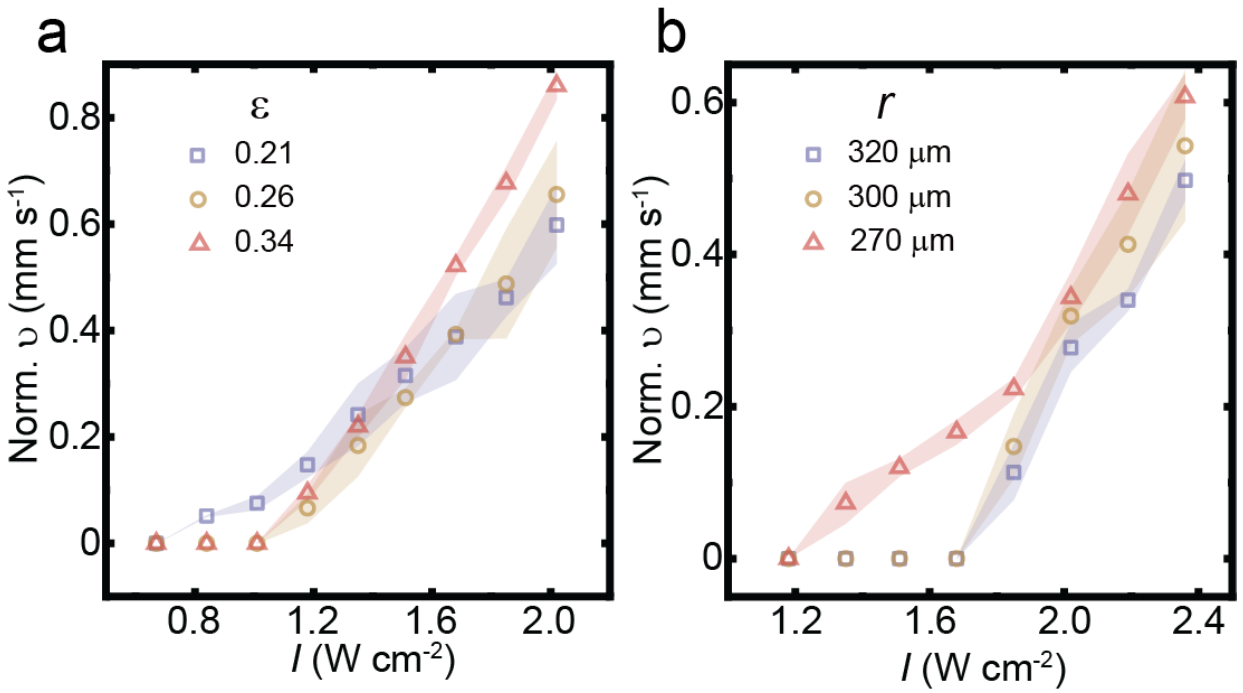

Supplementary Figure 31. Movement on a thread. Torus slenderness (a) and fiber radius (b) affect the normalized translation velocity υ on a thread in air. Irradiation conditions: 532 nm, different light intensities. Thread diameter: 50 μm. The error bars are displayed as mean values +/- standard deviation (n = 3). The same sample was measured repeatedly.

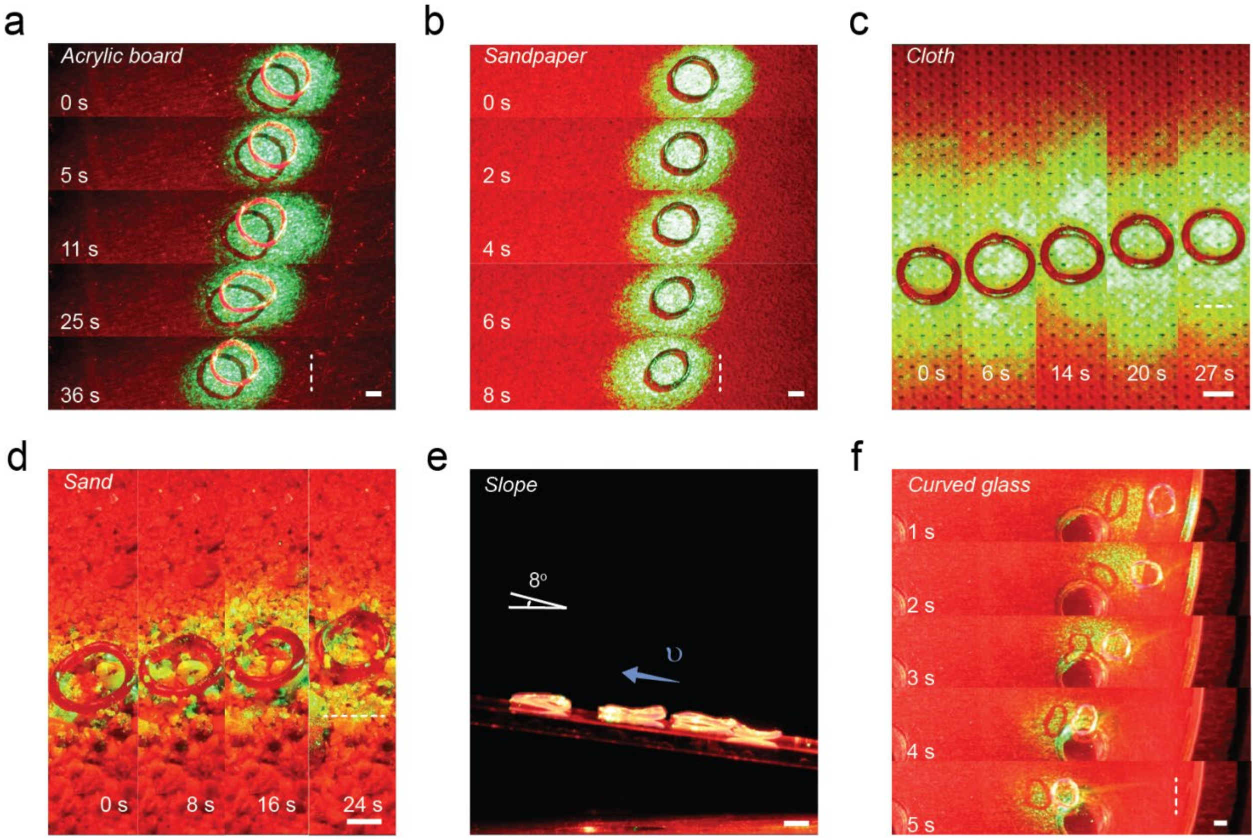

Supplementary Figure 32. Movement on various surfaces. Snapshots of active torus locomoting on (a) acrylic board, (b) sandpaper, (c) cloth, (d) sand, (e) glass slope, and (f) curved surface. Irradiation conditions: 532 nm 1.9 W cm^{-2}. Scale bars: 2 mm.

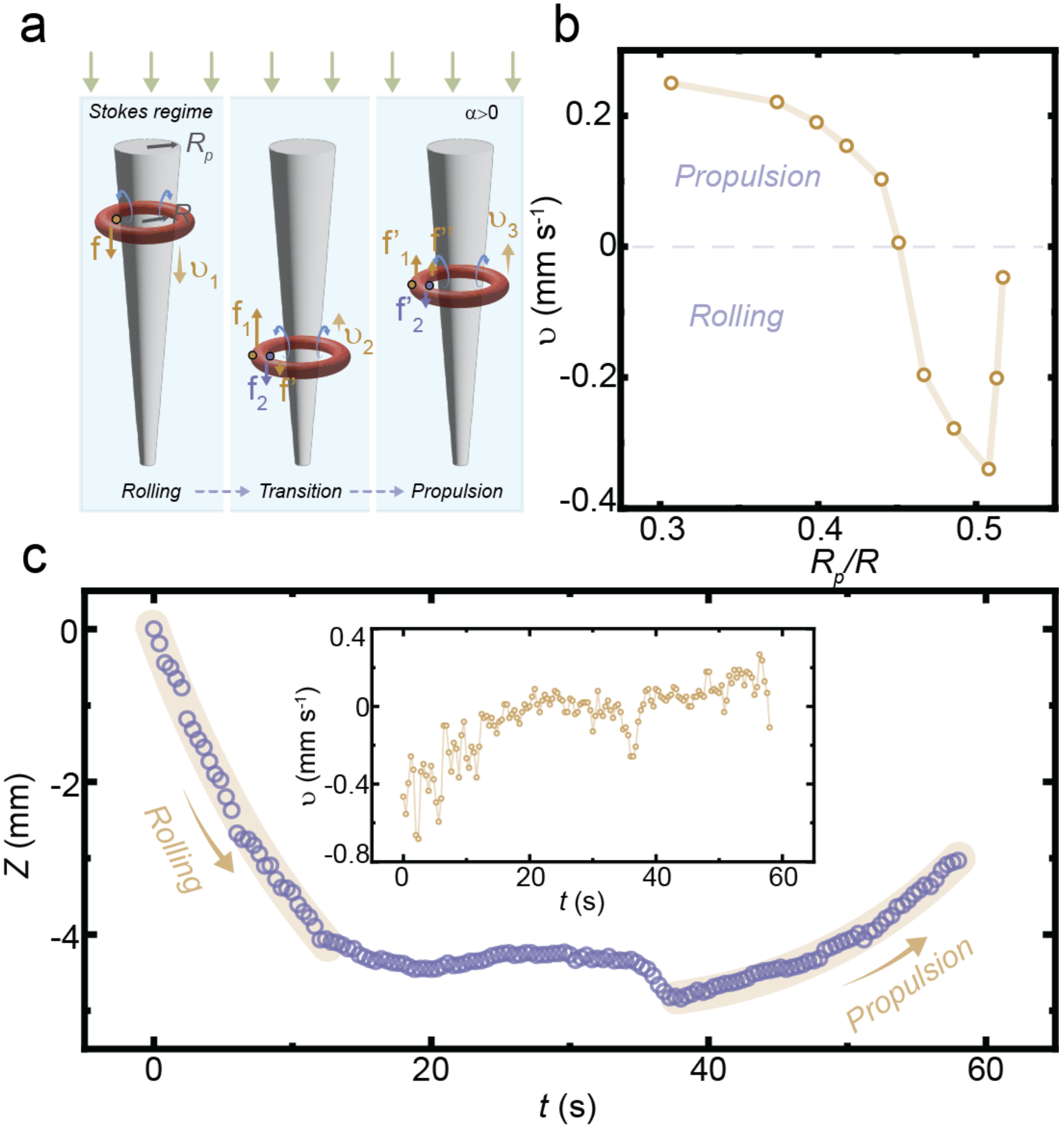

Supplementary Figure 33. Self-change of motion direction. (a) Schematics of a torus locomoting on a tapered pipette in Stokes regime, along with force analysis at different stages. (b)Variation of translational velocity with the change in aspect ratio between the pipette radius R_p and the radius of the LCE torus R. (c) Trajectory of the torus moving along the tapered pipette and corresponding temporal evolution of translational velocity (inset). Irradiation conditions: 532 nm, 7.3 W cm^{-2}.

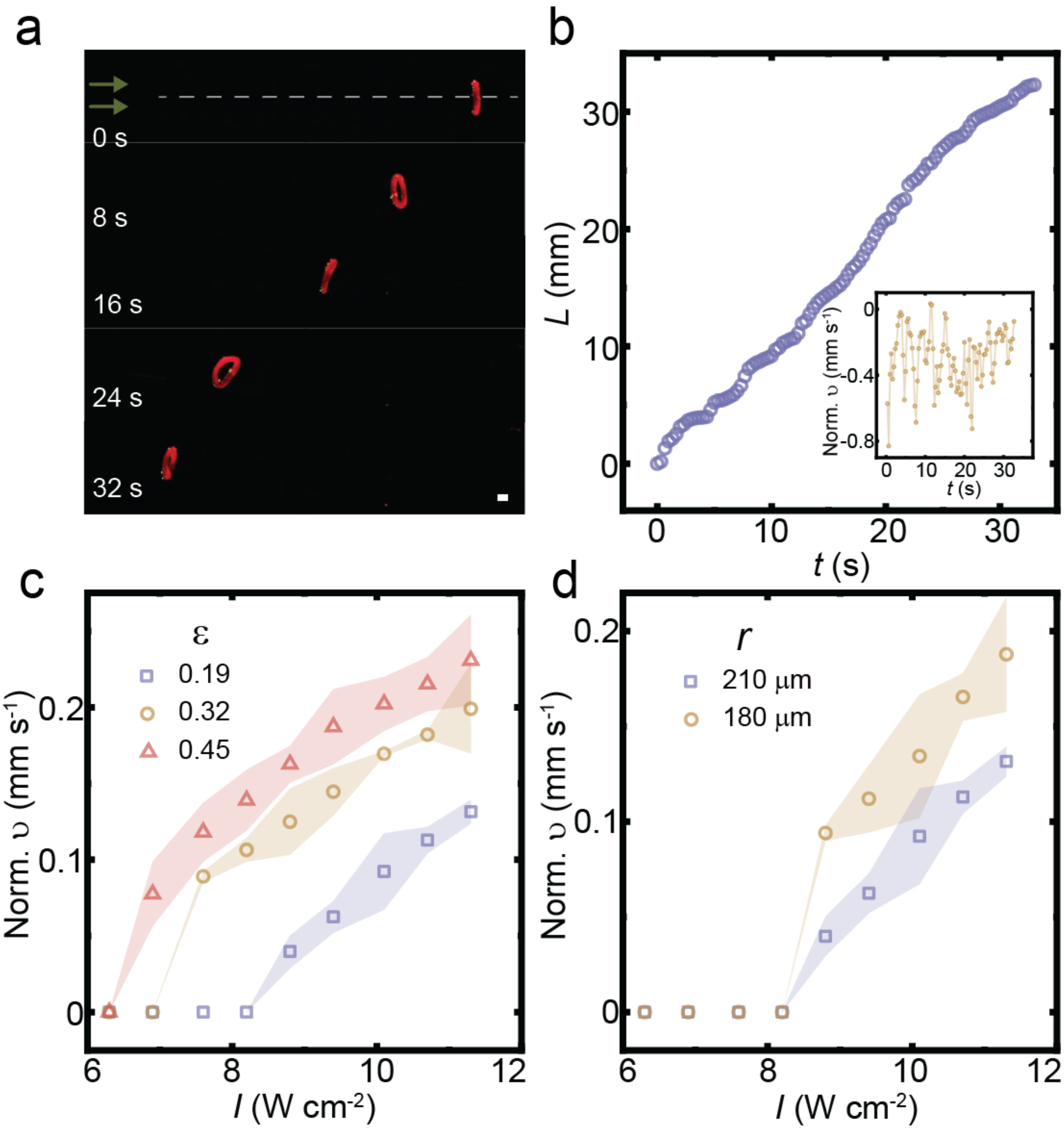

Supplementary Figure 34. Movement on a thread in glycerol. (a) Snapshots of active torus on thread in glycerol, and (b) the corresponding movement trajectories. The grey dashed line represents the thin thread. Inset: normalized translation velocity υ. Irradiation conditions: 532 nm, 7.3 W cm^{-2}. Scale bar: 1 mm. Torus slenderness (c) and fiber radius (d) affect the translation velocity on a thread in glycerol. Laser: 532 nm, different light intensities. Thread diameter $\phi = 50\ \mu$m. The error bars are displayed as mean values +/- standard deviation (n = 3). The same sample was measured repeatedly.

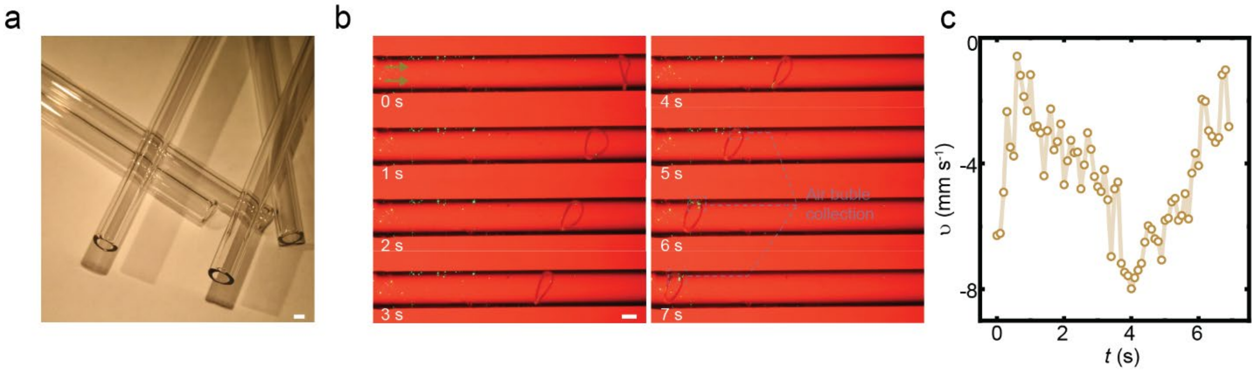

Supplementary Figure 35. Crawling in glass conduit. (a) Photograph of glass conduits. (b) Snapshots of active torus moving in a glass conduit filled with glycerol, enabling air bubble collection. (c) Corresponding translation velocity υ. Irradiation conditions: 532 nm, 7.3 W cm^{-2}. Scale bar: 2 mm.

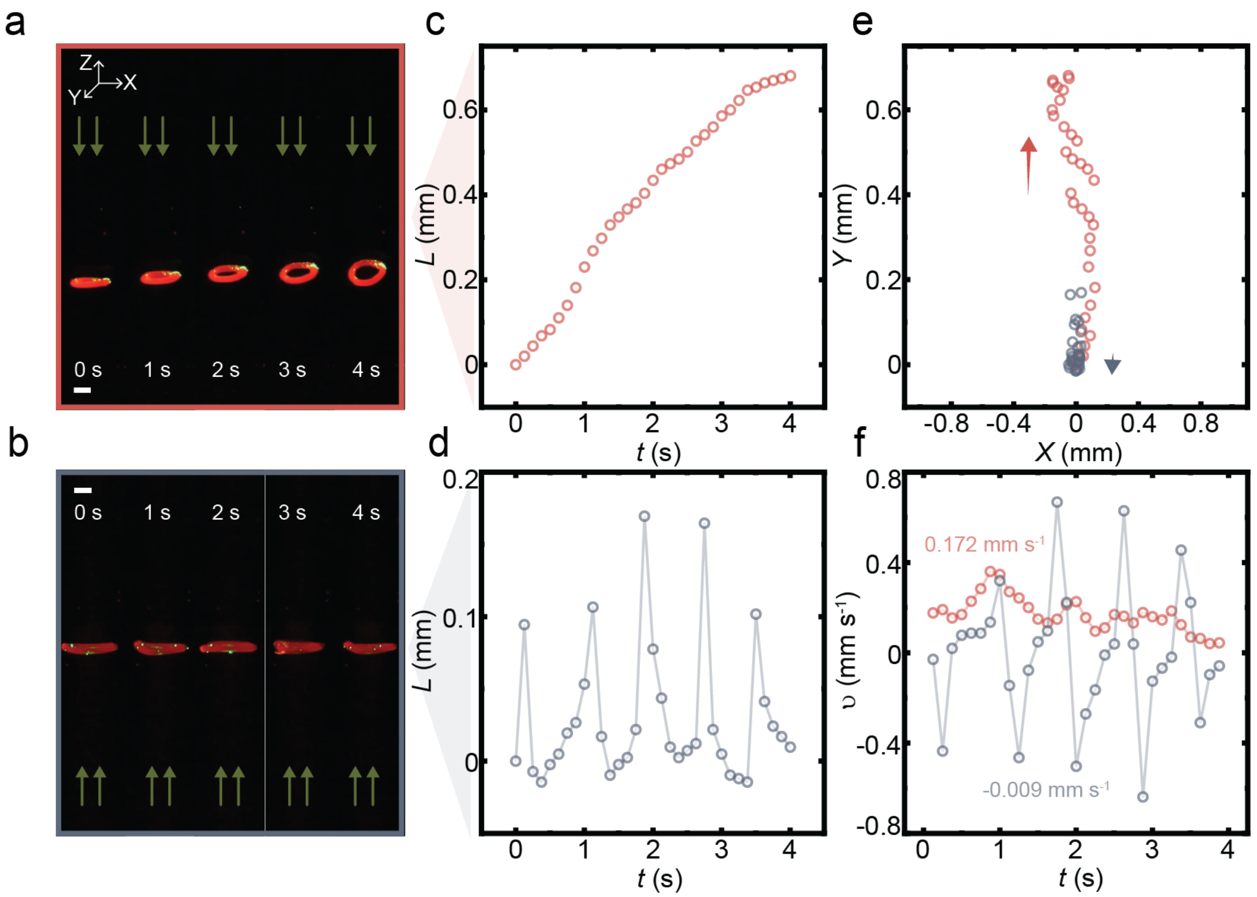

Supplementary Figure 36. Untethered swimming in glycerol. Snapshots of active torus swimming in glycerol in (a) upwards, (b) downwards directions and the corresponding trajectories (c) and (d). (e) Scheme concluding normalized courses for upwards and downwards swimming. The colored arrows indicate the moving directions in the two cases. (f) Corresponding translational velocity υ. The value suggests the average velocity, while the sign represents the movement direction. Irradiation conditions: 532 nm, 7.3 W cm^{-2}. Scale bars: 1 mm.

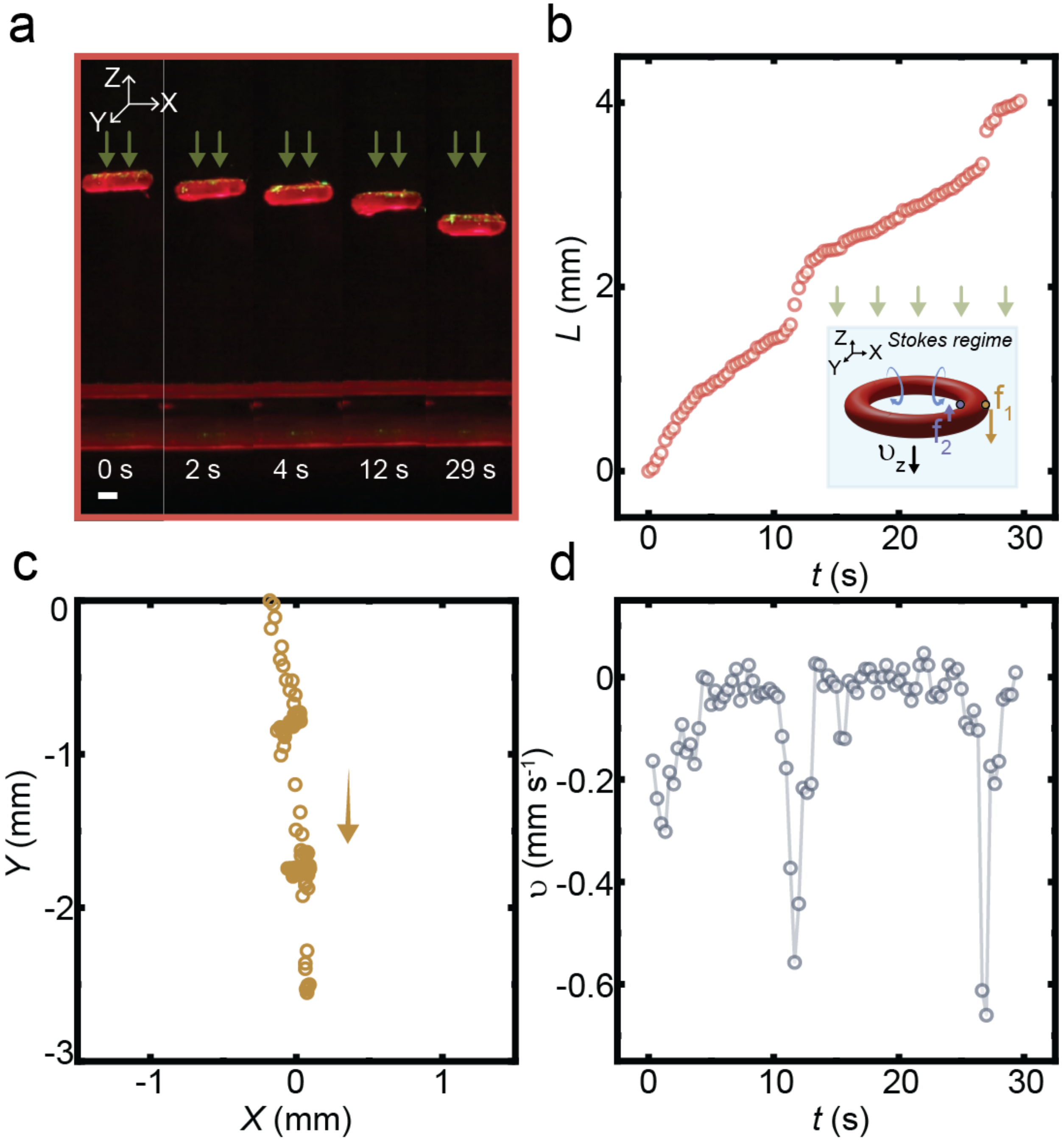

Supplementary Figure 37. Untethered swimming in Stokes regime. Snapshots of active torus ($\alpha < 0$) swimming downwards in PDMS when irradiated from the top and (b) corresponding movement trajectory and force analysis (inset). (c) The downwards swimming course in the X-Z plane and (d) corresponding temporal evolution of translational velocity υ along the Z axis. Irradiation conditions: 532 nm, 7.3 W cm^{-2}. Scale bar: 1 mm.

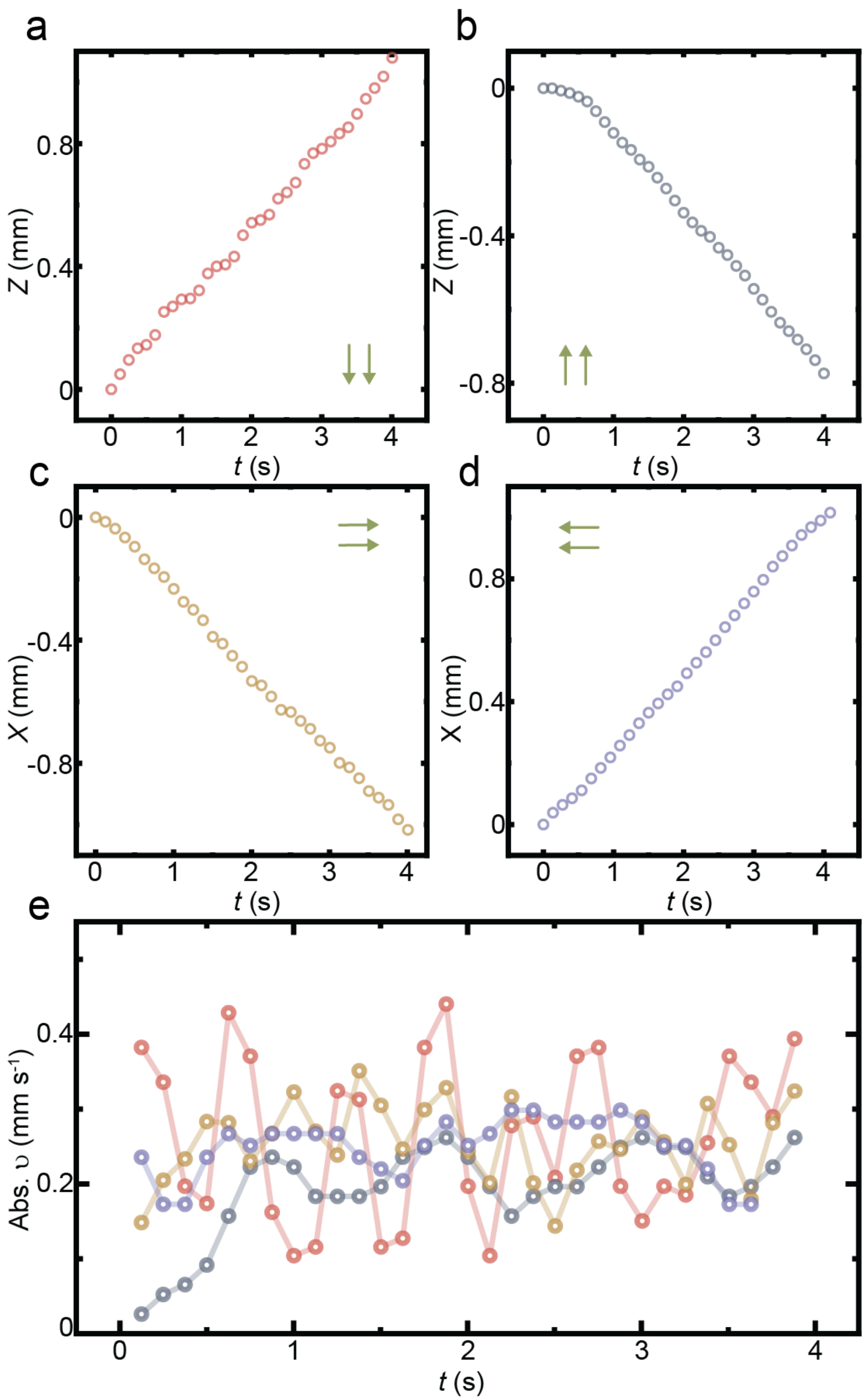

Supplementary Figure 38. Untethered swimming in Stokes regime. Individual swimming course in PDMS in (a) upwards, (b) downwards, (c) leftwards, and (d) rightwards direction. Green arrows represent the illumination direction. (e) Absolute translational velocity υ in four directions. Irradiation conditions: 532 nm, 7.3 W cm^{-2}.

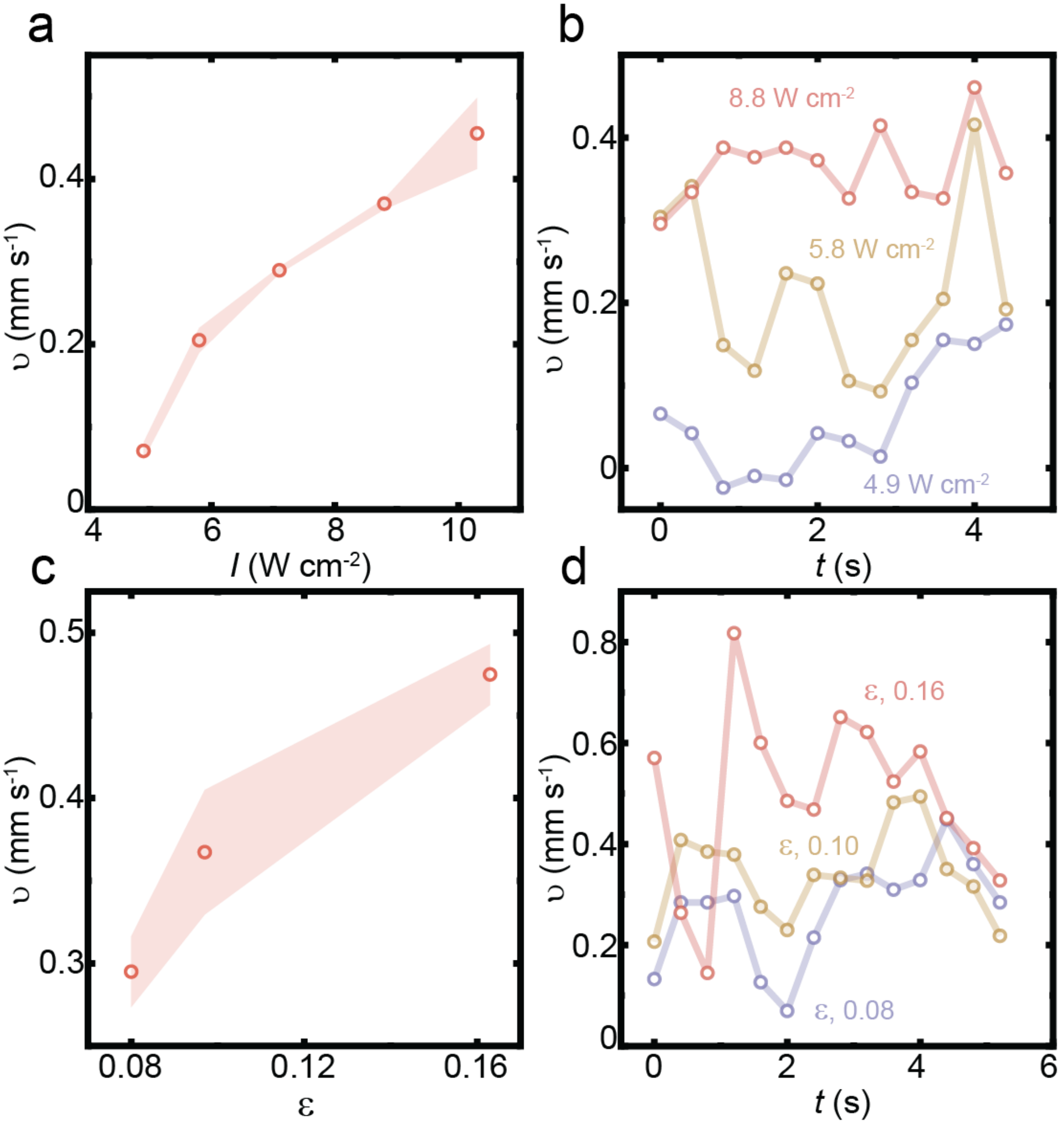

Supplementary Figure 39. Swimming velocity promotion. (a) Swimming velocity at various light intensities and (b) corresponding temporal evolution of the swimming velocity υ under 532 nm illumination. (c) Swimming velocity at varied slenderness and (d) corresponding temporal evolution swimming velocity υ. Irradiation conditions: 532 nm, 7.3 W cm^{-2}. The error bars are displayed as mean values +/- standard deviation (n = 3). The same sample was measured repeatedly.

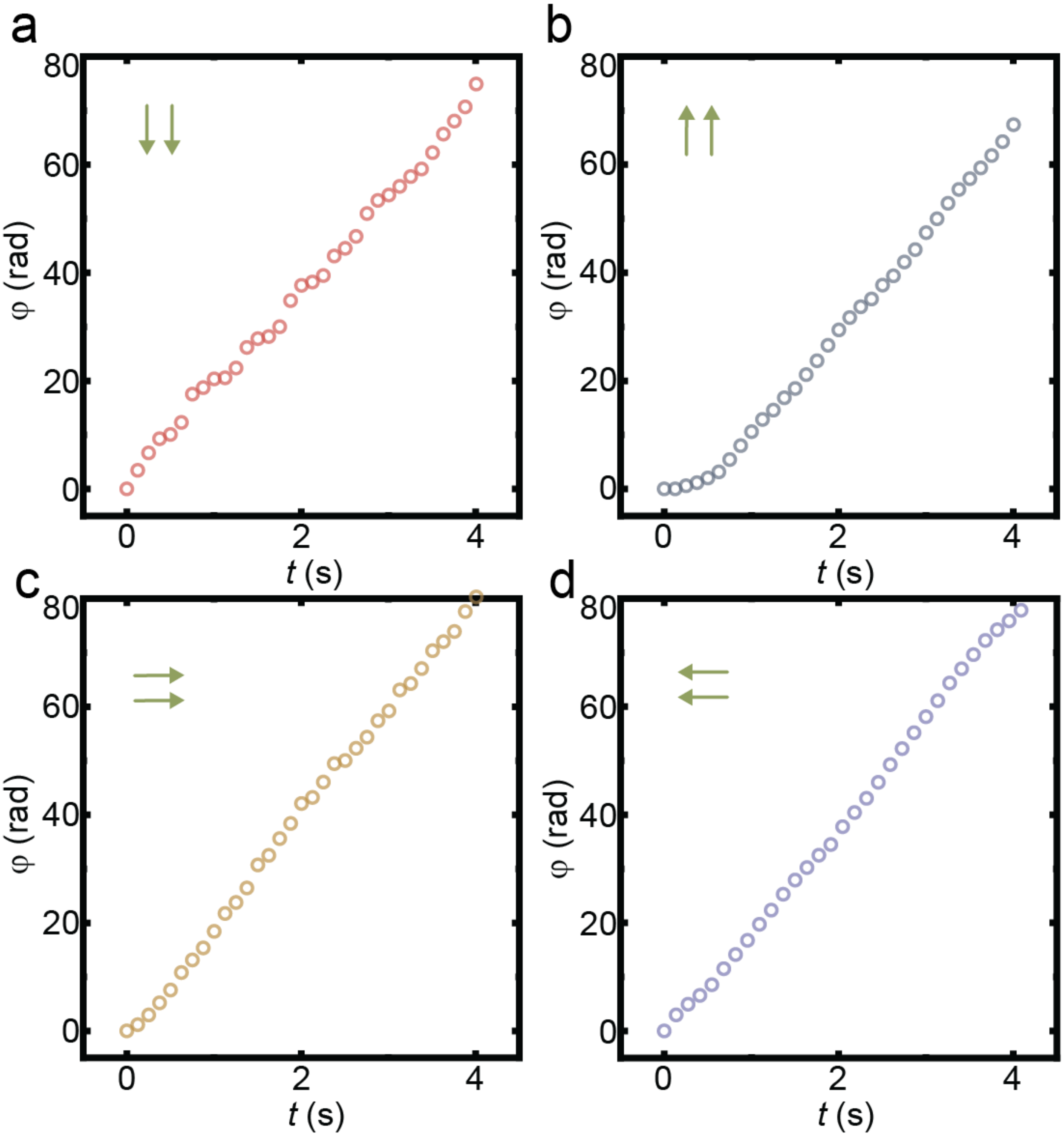

Supplementary Figure 40. Rotational angle in Stokes regime. Real-time individual rotational angle φ while swimming in PDMS in (a) upwards, (b) downwards, (c) leftwards, and (d) rightwards direction. Green arrows represent the illumination direction. Irradiation conditions: 532 nm, 7.3 W cm^{-2}.

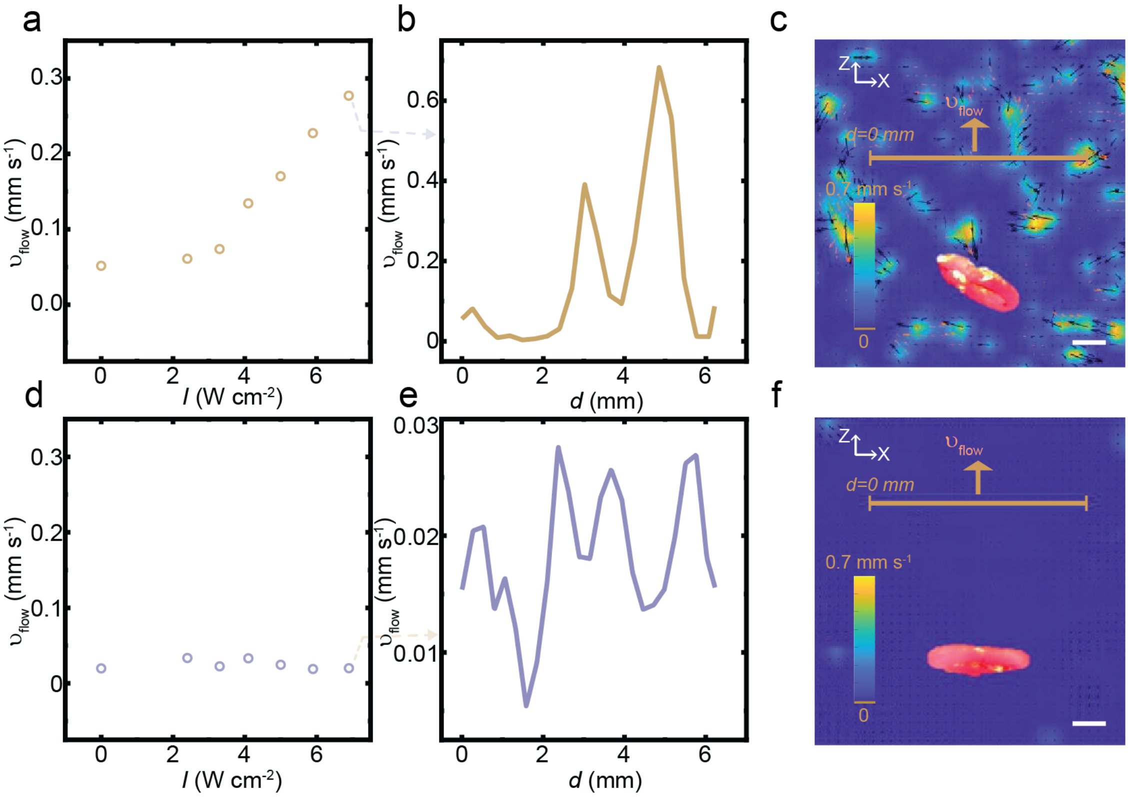

Supplementary Figure 41. Heat-induced convection. (a) Mean flow velocity along vertical direction, υ_{flow} upon different light intensities in glycerol. (b) υ_{flow} at the selected region inside glycerol. (c) PIV image of the photo-heat induced flow velocity field in glycerol. LCE fiber radius: 0.25 mm. Light: 532 nm, 7 W cm^{-2}. The horizonal line indicates the selected region for flow measurements. (d) υ_{flow} upon different light intensities inside non-polymerized PDMS. (e) υ_{flow} at the selected region in non-polymerized PDMS. (f) PIV image of the photo-heat induced flow field in non-polymerized PDMS. LCE fiber radius: 0.25 mm. Light: 532 nm, 7 W cm^{-2}. Scale bars: 1 mm. The liquids were mixed with 100 μm glass microspheres (Thermo scientific) for tracking the instantaneous velocity field.

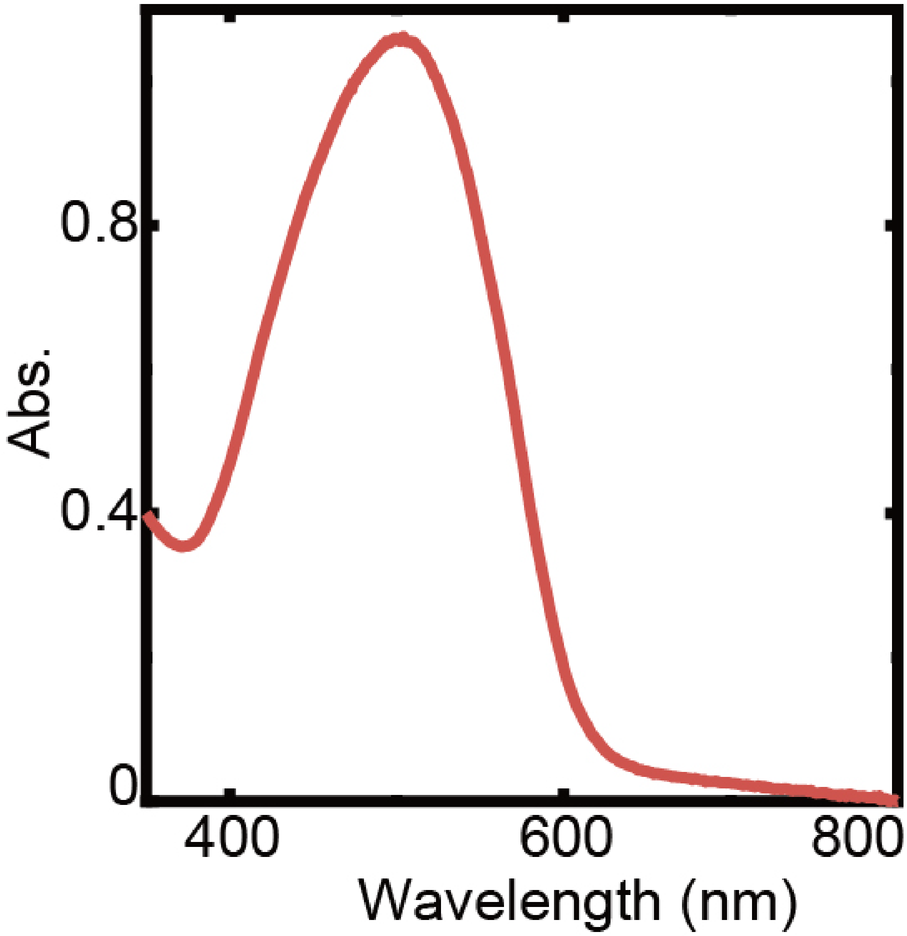

Supplementary Figure 42. Optical property. Absorption spectra of a 10-micron thick LCE film after dyeing.

3. Supplementary Video captions.

Supplementary Video 1-Thermally driven everting ZEEM torus.

This video shows the ZEEM torus ($\alpha < 0$) becomes active and starts to evert when placed on a hot plate at 120 °C. The rotation of black mark suggests the eversion event. Parameters of torus: fiber radius, 0.28 mm; torus radius, 2.52 mm.

Supplementary Video 2-Infrared imaging of optically driven inverting ZEEM torus under top-down illumination.

This video shows infrared temperature analysis of the ZEEM torus ($\alpha < 0$) inverting on dry land when receiving top-down illumination. Parameters of torus: fiber radius, 0.28 mm; torus radius, 2.52 mm. Irradiation conditions: 532 nm, 1.8 W cm^{-2}.

Supplementary Video 3-Optically driven rotation of ZEEM torus on air-glycerol interface.

This video shows the ZEEM torus ($\alpha < 0$) inverts on glycerol surface when receiving top-down illumination. Parameters of torus: fiber radius, 0.16 mm; torus radius, 2.80 mm. Irradiation conditions: 532 nm, 2.9 W cm^{-2}.

Supplementary Video 4-Terrestrial movement of ZEEM torus walking on land.

This video shows uni-directional movement of a ZEEM torus ($\alpha < 0$) on dry land upon oblique illumination. Parameters of torus: fiber radius, 0.38 mm; torus radius, 1.60 mm. Irradiation conditions: 532 nm, 1.9 W cm^{-2}.

Supplementary Video 5-Terrestrial movement of ZEEM torus crawling on a thread.

This video shows the ZEEM torus ($\alpha < 0$) moving on a thin thread under horizontal illumination in air. Parameters of torus: fiber radius, 0.30 mm; torus radius, 2.13 mm. Thread diameter: 50 μm. Irradiation conditions: 532 nm, 1.9 W cm^{-2}.

Supplementary Video 6-ZEEM torus climbing on a pipette in glycerol.

This video shows the ZEEM torus ($\alpha < 0$) climbing on a tapered pipette when receiving top-down light field in glycerol. Parameters of torus: fiber radius, 0.38 mm; torus radius, 2.36 mm. Irradiation conditions: 532 nm, 7.3 W cm^{-2}.

Supplementary Video 7-ZEEM torus moving in a glass conduit filled with glycerol.

This video shows the ZEEM torus ($\alpha > 0$) moving in a 5 mm glass conduit filled with glycerol. Parameters of torus: fiber radius, 0.23 mm; torus radius, 2.50 mm. Irradiation conditions: 532 nm, 7.3 W cm^{-2}.

Supplementary Video 8-Steerable untethered swimming of ZEEM torus in Stokes regime.

This video shows the ZEEM torus ($\alpha > 0$) swimming untetheredly in lateral and vertical directions in PDMS elastomer base, a Stokes regime. Parameters of torus: fiber radius, 0.17 mm; torus radius, 2.20 mm. Dynamic viscosity (μ) and density (ρ) of PDMS at 25 °C are 5.5 Pa s and 1.1 g cm^{-3}, respectively. Irradiation conditions: 532 nm, 7.3 W cm^{-2}.

Supplementary Video 9-Optical reorientation in Stokes regime.

This video shows the ZEEM torus ($\alpha > 0$) reorienting its excitation facet in PDMS elastomer base, a Stokes regime. Parameters of torus: fiber radius, 0.17 mm; torus radius, 1.76 mm. Dynamic viscosity (μ) and density (ρ) of PDMS at 25 °C are 5.5 Pa s and 1.1 g cm^{-3}, respectively. Irradiation conditions: 532 nm, 7.3 W cm^{-2}.

Supplementary Video 10-Untethered swimming through three-dimensional space in Stokes regime.

This video shows the ZEEM torus ($\alpha > 0$) navigating through three-dimensional space in PDMS elastomer base, a Stokes regime. Parameters of torus: fiber radius, 0.17 mm; torus radius, 1.76 mm. Dynamic viscosity (μ) and density (ρ) of PDMS at 25 °C are 5.5 Pa s and 1.1 g cm^{-3}, respectively. Irradiation conditions: 532 nm, 7.3 W cm^{-2}.

References:

1. Baumann, A. *et al.* Motorizing fibres with geometric zero-energy modes. *Nat. Mater.* **17**, 523–527 (2018).
2. Li, K., Chen, Z., Wang, Z. & Cai, S. Self-sustained eversion or inversion of a thermally responsive torus. *Phys. Rev. E* **103**, 033004 (2021).
3. Johnson, R. E. & Wu, T. Y. Hydromechanics of low-Reynolds-number flow. Part 5. Motion of a slender torus. *J. Fluid Mech.* **95**, 263–277 (1979).
